# Effect of Partial Silica Replacement with Carbon Black, Graphene, and Carbon Nanotubes on the Fatigue Performance and Ageing Behaviour of SBR Compounds

**DOI:** 10.3390/ma19163503

**Published:** 2026-08-18

**Authors:** Tomasz Gozdek, Julita Sadurska, Katarzyna Klajn, Dariusz M. Bieliński

**Affiliations:** Institute of Polymer & Dye Technology, Lodz University of Technology, 90-537 Lodz, Poland; 258905@edu.p.lodz.pl (J.S.); katarzyna.klajn@p.lodz.pl (K.K.); dariusz.bielinski@p.lodz.pl (D.M.B.)

**Keywords:** styrene-butadiene rubber (SBR), carbon black, graphene, carbon nanotubes, fatigue resistance, dynamic ageing, crosslink density, microhardness, GC–IMS

## Abstract

This study investigated the effect of the partial replacement of silica with carbon fillers, namely carbon black (CB), graphene, and carbon nanotubes (CNTs), on the curing behaviour, thermo-mechanical properties, ageing resistance, and degradation of styrene-butadiene rubber (SBR) vulcanizates. The aim was to determine whether small amounts of carbon fillers could improve the durability-related properties of silica-filled SBR compounds. Graphene and CNTs reduced the maximum curing torque compared with the CB-filled compound while maintaining satisfactory curing characteristics. Thermal conductivity increased with temperature for all materials and reached 0.126 W·m^−1^·K^−1^ for the graphene-filled vulcanizate at 40 °C, compared with 0.109 and 0.107 W·m^−1^·K^−1^ for the CNT- and CB-filled compounds, respectively. Dynamic ageing tests revealed significant differences in self-heating behaviour. After 10,000 De Mattia cycles, the graphene-filled compound exhibited the lowest temperature increase (1.5 °C), whereas the CB5/Sil15 formulation showed the highest value (4.5 °C). GC-IMS analysis confirmed the formation of volatile degradation products during cyclic ageing, while increasing carbon filler content reduced the intensity of the characteristic VOC signals. Analysis of the crosslink structure indicated that CNT-containing compounds exhibited the highest resistance to ageing-induced structural changes, whereas graphene promoted an increase in the proportion of monosulfidic and carbon–carbon crosslinks. Overall, the results demonstrate that partial replacement of silica with carbon nanofillers improves the thermo-mechanical stability of SBR vulcanizates. Graphene provided the greatest enhancement in thermal conductivity and the lowest heat build-up, while CNTs showed the highest resistance to structural changes during dynamic ageing.

## 1. Introduction

By definition, material fatigue is the process of crack initiation and propagation caused by cyclic stresses that accumulate, particularly at the tips of pre-existing defects. Such defects may arise from poor dispersion of compound constituents, impurities present in the material, or damage introduced during processing and service. Owing to the pronounced hysteresis of rubber materials and their susceptibility to environmental factors such as oxygen and ozone, fatigue processes in elastomers are more complex than those observed in many conventional engineering materials [1].

The study of fatigue in rubber materials is of considerable importance because many elastomeric components are subjected to cyclic loading during service, which may ultimately lead to failure. Fatigue is associated with the initiation and subsequent propagation of cracks under repeated loading conditions. Cracks may develop even in regions that appear free of visible defects and propagate until catastrophic failure occurs. Therefore, identifying crack initiation sites is one of the key aspects of fatigue life assessment [1,2].

Depending on service conditions, rubber materials may be exposed to different loading modes, including tension–tension, tension–compression, tension–torsion, compression–torsion, and compression–compression [2]. In laboratory investigations, fatigue is commonly evaluated under either constant-amplitude or variable-amplitude loading. Among the factors affecting the fatigue behaviour of elastomers, strain-induced crystallization is particularly important, while the type of rubber and its mechanical properties also have a significant influence on fatigue performance [2,3].

The mechanical performance and fatigue resistance of rubber materials can be improved through appropriate compound formulation, particularly through the selection of suitable reinforcing fillers. Carbon black remains the most widely used reinforcing filler in the rubber industry because it significantly improves the mechanical properties and fatigue resistance of rubber vulcanizates [4,5]. In recent years, increasing attention has also been devoted to carbon-based nanofillers, especially graphene, owing to their potential to enhance the mechanical performance and durability of polymer composites [6]. When evaluating the fatigue behaviour of rubber materials, the effects of cyclic deformation should also be considered, since repeated loading results in hysteretic energy dissipation, which contributes to material degradation and may reduce the service life of elastomeric components [2,3].

Over the past several decades, numerous studies have investigated the fatigue behaviour of rubber materials to better understand the mechanisms responsible for crack initiation and propagation during cyclic loading. These studies have demonstrated that fatigue life strongly depends on compound composition, particularly on the type and content of reinforcing fillers and the nature of filler–rubber interactions [2,3,4,5]. Properly selected reinforcing fillers can delay crack initiation and propagation, improve the mechanical properties of vulcanizates, and enhance their resistance to cyclic loading [4,5,6]. However, several studies have also indicated that excessive filler loading may promote agglomeration, deteriorate filler dispersion, and consequently reduce fatigue performance [4,5]. In addition, stress softening (the Mullins effect) and hysteresis associated with cyclic loading are important phenomena influencing the durability of elastomeric materials [2,3].

Despite numerous studies on carbon black- and nanocarbon-filled elastomers, the mechanisms by which different carbon fillers influence fatigue-induced degradation and possible network reorganization are still not fully understood. In particular, there is limited knowledge regarding the relationship between dynamic ageing, changes in crosslink structure, and the formation of volatile degradation products. Therefore, this work investigates the effect of partially replacing silica with carbon black, graphene, and carbon nanotubes on the properties of SBR compounds. The study focuses on curing behaviour, mechanical performance, thermal conductivity, fatigue resistance, and structural changes occurring during cyclic loading. Furthermore, an attempt was made to correlate changes in crosslink density and microhardness with the volatile compounds released during dynamic ageing, as identified by GC-IMS analysis.

## 2. Materials and Methods

### 2.1. Formulations

The polymer matrix used in the prepared compounds was styrene-butadiene rubber (SBR). The curing system consisted of sulfur, stearic acid, zinc oxide, and TBBS (N-tert-butylbenzothiazole-2-sulfenamide). Compounds filled with silica, carbon black (at various loadings), graphene, and carbon nanotubes were prepared. An efficient sulfur curing system was applied during compounding. The formulations of the prepared compounds are presented in Table 1.

For the comparative study of different carbon fillers, the loading of graphene, carbon nanotubes, and carbon black was fixed at 1 phr while maintaining a constant total filler content of 20 phr. This experimental design enabled a direct comparison of the effect of filler type under identical formulation conditions, whereas determining the optimum loading for each filler was beyond the scope of the present study.

### 2.2. Preparation of Blends

#### 2.2.1. Preliminary Mixing

Rubber and all additives, except for sulfur, were mixed in a Brabender N50 laboratory internal mixer (Brabender^®^ GmbH & Co. KG, Duisburg, Germany) at a rotor speed of 20 rpm.

#### 2.2.2. Final Blending

Following preliminary mixing, five batches of each formulation were combined using a David Bridge laboratory two-roll mill (David Bridge, Southampton, UK). This step was performed to further improve the dispersion of components that had not been completely dispersed during mixing in the internal mixer. The mill was equipped with two rolls, each 300 mm long and 150 mm in diameter. The friction ratio between the rolls was 1.1, corresponding to rotational speeds of approximately 20 and 18 rpm, respectively. Sulfur was incorporated during this stage of processing.

### 2.3. Curing Kinetics

The curing characteristics of each compound were determined using an MDR-2000 rheometer (Alpha Technologies, Hudson, OH, USA) at 170 °C, in accordance with ISO 6502-1:2025 [7]. Each measurement was conducted for 60 min. The oscillation frequency was 1.667 Hz, and the oscillation amplitude was 3°. All measurements were performed in triplicate, with deviations between individual measurements not exceeding 5%. The minimum torque (Mmin), maximum torque (Mmax), and torque increment (ΔM) were calculated according to the following equation:

(1) $$∆M=\;M_{max}-M_{min}$$

where:

ΔM—torque increase during compound vulcanization [dNm];

M_min_—minimum torque during compound vulcanization [dNm];

M_max_—maximum torque during compound vulcanization [dNm].

### 2.4. Vulcanisation Process

Vulcanization of mixtures.

#### 2.4.1. Using a Larger Laboratory Press

The compounds were vulcanized in a Nysa laboratory press (ZUP, Nysa, Poland) at 170 °C and 100 MPa.

#### 2.4.2. Using a Smaller Laboratory Press

**Figure 1:**
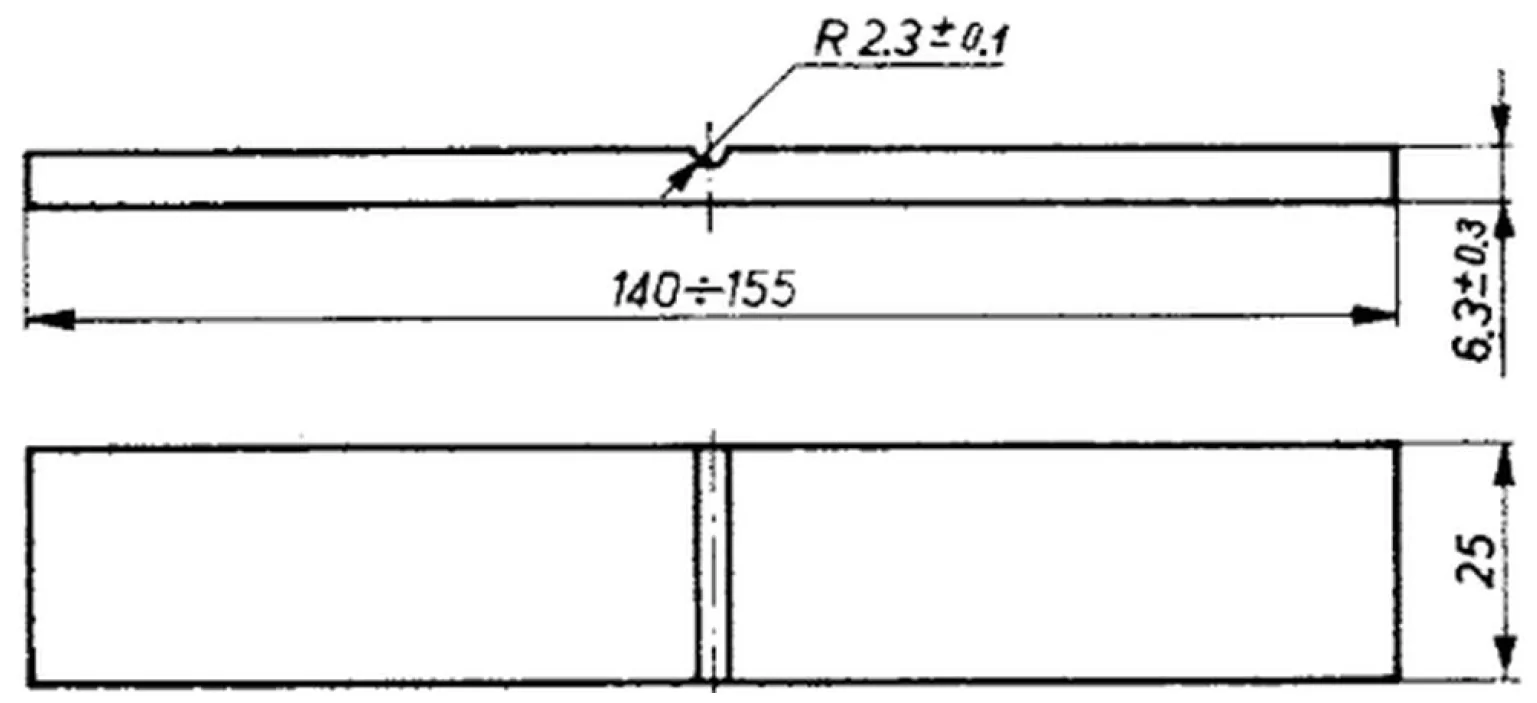
Shape and dimensions of a sample characteristic for testing with the de Mattia apparatus in accordance with PN-86/C-04247 [8].

Specimens intended for cyclic fatigue testing (Figure 1) were vulcanized using a Skamet 54436 (SKAMET, Skarżysko-Kamienna, Poland) laboratory press.

Each of the mixtures was vulcanized at a temperature of 170 °C and a pressure of 100 MPa.

### 2.5. Tensile Strength and Hysteresis

Tensile properties were determined in accordance with ISO 37 [9] using a Zwick/Roell 1435 universal testing machine (Zwick/Roell, Ulm-Einsingen, Germany). Five independent measurements were performed for each compound. The Mullins effect was calculated from the hysteresis data using the following equation:

(2) $$E_{m}=\frac{W_{1}-W_{5}}{W_{5}}\;*\;100\%$$

where:

E_m_—Mullins effect [%];

W_1_—hysteresis losses during the first stretching of the sample [Nmm];

W_5_—hysteresis losses during the fifth stretching of the sample [Nmm].

### 2.6. Dynamic Mechanical Analysis

Dynamic mechanical analysis (DMA) was performed in tensile mode using a DMA/SDTA861e analyzer (Mettler Toledo, Greifensee, Switzerland). The storage modulus (E′) and loss modulus (E″) were recorded over a temperature range from −100 °C to 80 °C at a heating rate of 3 °C/min. During the measurements, the specimens were subjected to cyclic deformation with an amplitude of 10 μm and a frequency of 1 Hz. Three independent measurements were performed for each compound.

### 2.7. Payne Effect

Dynamic mechanical measurements were performed using an Ares G2 rheometer (TA Instruments, New Castle, DE, USA). The Payne effect was evaluated by means of a strain sweep test carried out at a constant angular frequency of 10 rad/s. The shear strain amplitude was varied from 0.1% to 150%. Three independent measurements were performed. The Payne effect (ΔG′) was calculated according to the following equation:

(3) $$∆G^{\prime }\;=\;{∆G^{\prime }}_{0}-{∆G^{\prime }}_{\infty }$$

where:

G′_0_—value of the conservative modulus for the lowest applied shear deformation amplitude;

G′∞—value of the conservative modulus for the highest applied shear deformation amplitude.

### 2.8. Thermal Conductivity Test

Thermal conductivity was measured in accordance with ISO 8301 [10] using specimens with dimensions of 20 cm × 20 cm × 2 cm. Measurements were performed using a Netzsch HFM 446 Lambda heat flow meter (Netzsch, Selb, Germany). A compressive load of 2 kPa was applied throughout the test. Thermal conductivity was determined at three temperature levels: 10 °C, 20 °C, and 40 °C. For each specimen, measurements were conducted at six different locations, with 15 measurements performed at each location.

### 2.9. Rubber Fatigue

The De Mattia fatigue testing apparatus, compliant with PN-86/C-04248, was used not to perform a conventional rubber fatigue test but as a device for controlled cyclic bending of specimens prior to further analyses.

**Figure 2:**
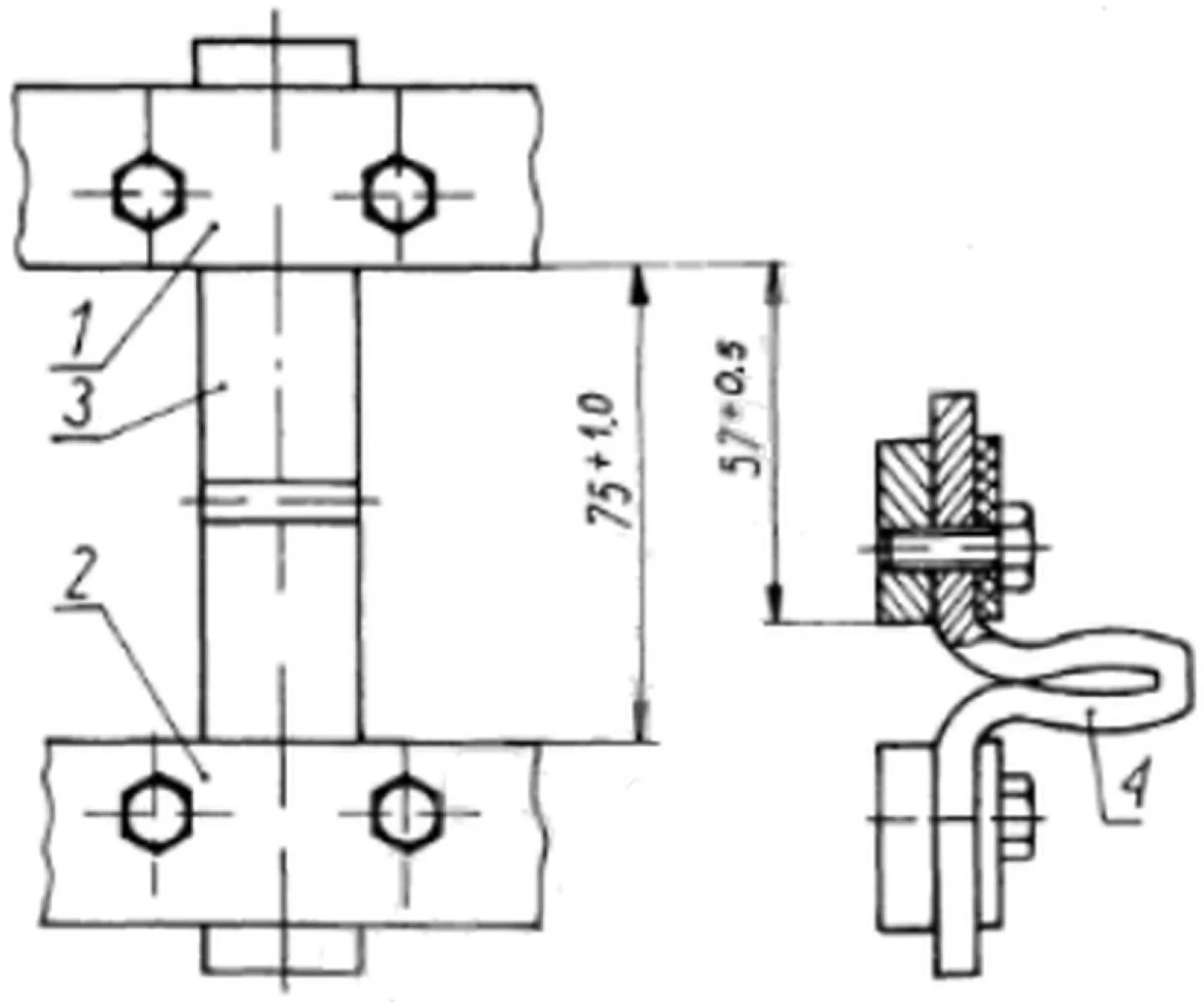
Placement of the sample in the holders of the de Mattia apparatus in accordance with PN-86/C-04248 [8]: 1—top handle, 2—lower handle, 3—test sample, 4—test sample in the maximum bend position.

The specimens were subjected to repeated bending at a frequency of 5 Hz. The apparatus consists of two parallel bars equipped with specimen holders. The lower bar is driven by a motor through an eccentric mechanism, producing 300 ± 10 vertical strokes per minute, while the upper bar remains stationary throughout the procedure (Figure 2).

Unlike the standard De Mattia test, which is designed to initiate and monitor crack propagation until specimen failure, the present study did not evaluate the fatigue resistance of the material. Instead, the test parameters were selected to expose the specimens to repeated bending without causing failure. The objective was to obtain an intact central section of the specimen after a predetermined number of bending cycles for subsequent material characterization.

### 2.10. Thermographic Analysis During Cyclic De Mattia Fatigue Testing

Infrared thermography was used to monitor the temperature evolution of rubber compounds during cyclic De Mattia fatigue testing. Prior to testing, a thermal imaging camera (VIGOcam V50, VIGO System S.A., Ożarów Mazowiecki, Poland) was positioned approximately 1 m in front of the testing machine to ensure a full view of the specimen throughout the experiment. The emissivity of the rubber surface was set to 0.95. Fatigue tests were interrupted after 100, 500, 2000, and 10,000 loading cycles. Thermal images were acquired immediately before the start of the test and directly after completion of each loading interval. Measurements were performed on three to four specimens for each compound. The temperature rise (ΔT) was determined as the difference between the maximum surface temperature measured after cyclic loading and the initial temperature recorded before the test. This procedure enabled the assessment of heat generation associated with internal energy dissipation during cyclic deformation and provided indirect information on the influence of filler composition on hysteresis and thermo-mechanical behaviour.

### 2.11. Crosslinking Density

#### 2.11.1. Equilibrium Swelling Method

**Figure 3:**
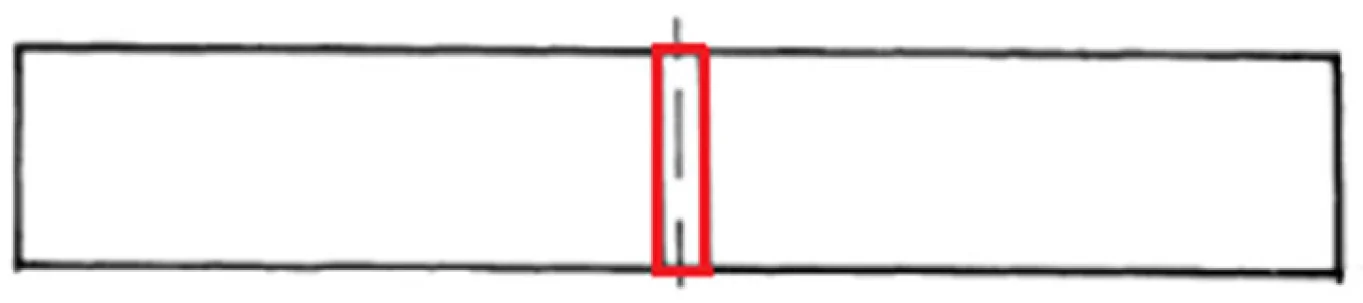
The shape of the sample subjected to dynamic aging testing; the area marked in red is used for further testing.

Specimens were cut from the vulcanizates, as illustrated in Figure 3. Test pieces weighing 20–50 mg were prepared, immersed in toluene, and subjected to equilibrium swelling measurements in accordance with ISO 1817:2026 [11]. For each specimen, five test pieces were analysed, and the standard deviation of the obtained results did not exceed 5%. The equilibrium volumetric swelling (Q_v_) was calculated according to the following equation:

(4) $$Q_{v}=\;Q_{w}\;*\;\left(\frac{d_{k}}{d_{r}}\right)$$

where:

Q_w_—equilibrium weight swelling;

d_k_—rubber density in the vulcanizate;

d_r_—solvent density, for toluene equal to: d_toluene25_ = 0.862 g/cm^3^ [12].

The equilibrium weight swelling Q_w_ was calculated using the following formula:

(5) $$Q_{w}=\;\frac{\left(m_{sp}-m_{s}\right)}{m_{s}}\;$$

where:

m_sp_—mass of the swollen sample;

m_s_—mass of the dry sample (after swelling and drying to the so-called dry mass).

If the vulcanizate contains mineral substances, the equilibrium weight swelling is calculated using the formula:

(6) $$Q_{w}=\;\frac{\left(m_{sp}-m_{s}\right)}{m_{s^{*}}}$$

where:

(7) $$m_{s}\;*=m_{s}-\;m_{0\;}\left(\frac{m_{n}}{m_{c}}\right)$$

where:

m_s*_—mass of the sample dried after swelling, adjusted for the proportion of mineral substances;

m_0_—initial mass of the sample;

m_n_—mass of mineral substances contained in the vulcanizate;

m_c_—total mass—mass of all components of the mixture.

#### 2.11.2. OTAM-OTAT Thiol-Amine Analysis Method

This method employs two thiol-amine reagents, referred to as soft and hard reagents.

The soft thiol-amine reagent (OTAM) was prepared by mixing 0.4 mol of isopropyl mercaptan and 0.4 mol of piperidine in toluene. To prepare 0.5 dm^3^ of solution, 18.72 cm^3^ of isopropyl mercaptan and 19.78 cm^3^ of piperidine were used, and the volume was adjusted with toluene.

The hard thiol-amine reagent (OTAT) was prepared by mixing 1.0 mol of dodecyl mercaptan with piperidine. To prepare 0.5 dm^3^ of solution, 119.77 cm^3^ of dodecyl mercaptan was used, and the volume was adjusted with piperidine.

The specimens used for the OTAM-OTAT analysis were prepared in the same manner as those used for the equilibrium swelling measurements. For each specimen, five test pieces were analysed, and the standard deviation of the obtained results did not exceed 10%.

Prior to the analysis, the specimens were swollen in toluene for 12 h. Subsequently, they were treated with the OTAM and OTAT reagents under an argon atmosphere. The treatment time was 2 h for OTAM and 24 h for OTAT. After treatment, the specimens were washed several times with toluene to remove residual reagents. They were then placed in weighing vessels, immersed in toluene, and conditioned at 296 K for 48 h.

After conditioning, the specimens were removed from the solvent, briefly rinsed with diethyl ether, and weighed. Subsequently, they were dried at 333 K for 48 h and weighed again after reaching constant mass.

Because the initial sample mass (m_0_) was not available, the calculation of equilibrium weight swelling (Qw) after treatment with the OTAM or OTAT reagents was based on an approximate equation, which introduced a small error into the final result. Using the obtained volumetric swelling value (Qv), the volume fraction of rubber in the swollen specimen (Vr) was calculated according to the following equation.

The crosslink density (n) was then determined using the Flory-Rehner equation [13]:

(8) $$n=-\left[\frac{ln\left(1-V_{r}\right)+\;V_{r}+m\;*\;\;V_{r}^{2}}{V_{0}\left(V_{r}^{\frac{1}{3}}-2*\frac{V_{r}}{f}\right)}\right]$$

where:

f—network functionality, f = 4;

V_0_—molar volume of the solvent, for toluene equal to: V_0 toluene_ = 106.893 cm^3^/mol;

χ—Huggins parameter (concerning cross-linked rubber–solvent interactions; characteristic for a given rubber–solvent pair):

(9) $$\chi =\;\chi_{0}+\;\beta \;*\;\;V_{r}$$

χ_0_, β—parameters of the straight line equation.

After obtaining the crosslink density, the concentration of network nodes (N) in the crosslinked sample, swollen only in toluene after treatment with the OTAM or OTAT solution, was then calculated. The following formula was used for this purpose:

(10) $$N=\;\frac{2n}{f}=\;\frac{n}{2}=\frac{1}{\left(2M_{c}\right)}$$

where:

(11) $$M_{c}=\;\frac{d_{k}}{n}$$

where:

n is expressed in mol/cm^3^;

M_c_—numerical average molecular weight of chains between network nodes [g/mol].

### 2.12. Microhardness

Microhardness measurements were performed on 2 mm thick specimens cut from the vulcanizates, as shown in Figure 3. A NanoTest 600 instrument (Micromaterials Ltd., Wrexham, UK) equipped with a Berkovich diamond indenter was used for this purpose. The indenter had the geometry of a three-sided pyramid with an equilateral triangular base and a vertex angle of 135°. Loading and unloading were carried out at a rate of dP/dt = 0.01 mN/s. The maximum indentation depth was set to 3500 nm. During the measurements, the temperature was maintained at 25 ± 2 °C and the relative humidity at 60 ± 5%. Ten independent measurements were performed for each specimen, and the deviations between the obtained results did not exceed 3%.

### 2.13. Analysis of Volatile Compounds Released After Aging

To investigate the volatile compounds released during the ageing process, GC-IMS measurements were performed using a coupled gas chromatography–ion mobility spectrometry system (MCC-IMS, G.A.S., Dortmund, Germany). Specimens previously subjected to cyclic fatigue testing in the De Mattia apparatus were used for the analysis. The central regions of the specimens were cut out, as shown in Figure 3, and subsequently divided into strips approximately 2 mm wide.

Samples weighing up to 10 g were placed individually in gas-tight 10 mL glass vials. Prior to analysis, each vial was conditioned at 30 °C for 5 min. The headspace gas was then introduced into the GC-IMS system using a sampling needle connected to the instrument.

Following injection, volatile compounds were first separated on a capillary gas chromatography column and subsequently analyzed using ion mobility spectrometry. Three independent measurements were performed for each specimen. The obtained spectra were used for the qualitative and semi-quantitative characterization of volatile compounds generated during dynamic ageing.

## 3. Results

### 3.1. Study of the Effect of Carbon Black Added in Various Quantities

#### 3.1.1. Curing Kinetics

**Figure 4:**
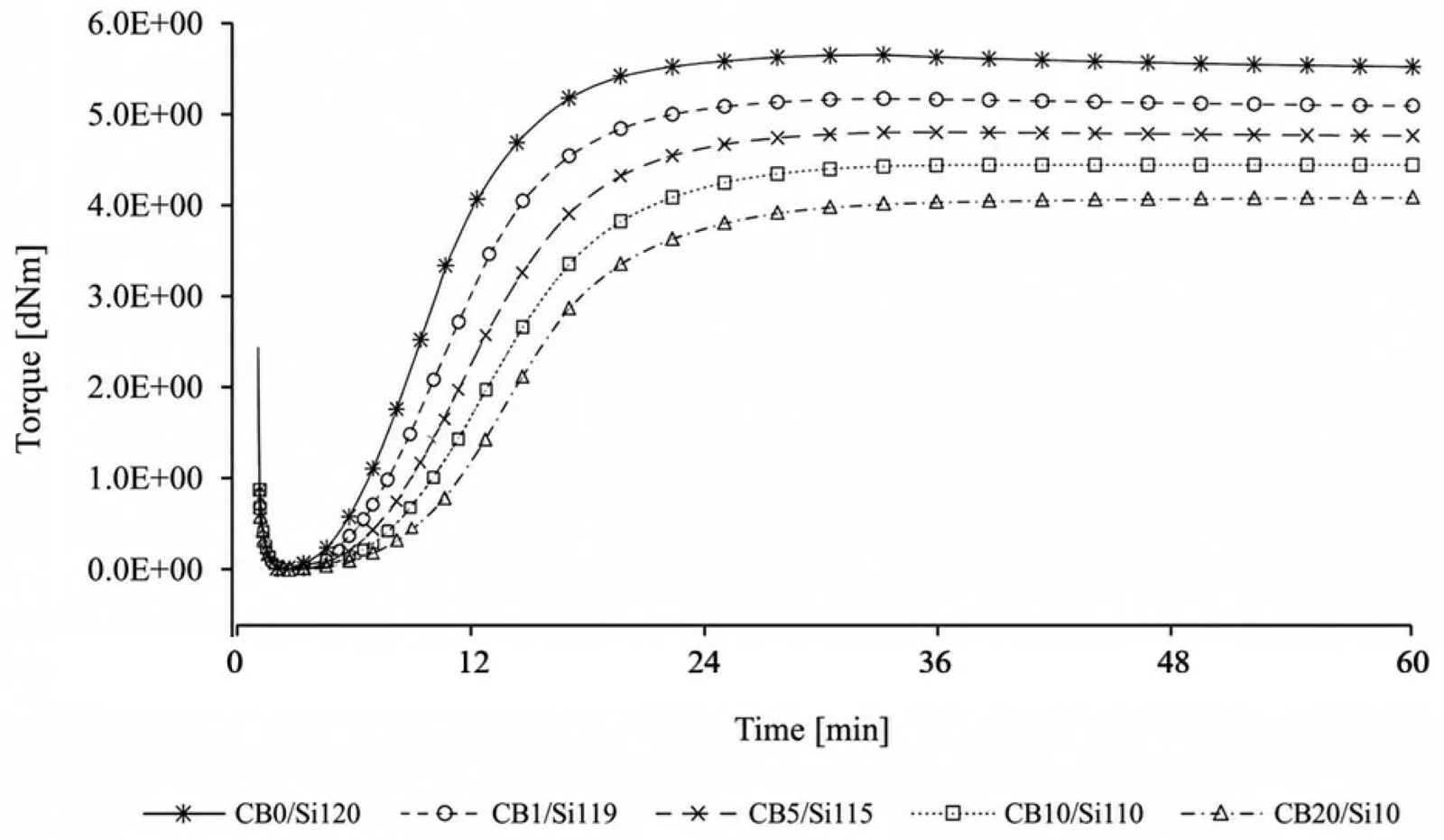
Curing kinetics of carbon black-filled SBR compounds.

An increase in carbon black content resulted in a higher final torque during curing (Figure 4). This effect can be attributed to the increased viscosity of the compound and the formation of a stronger filler network within the polymer matrix. Consequently, the proportion of filler–filler interactions increased, leading to a higher amount of bound rubber. It should also be noted that carbon black tends to form aggregates more readily than silica, particularly in the absence of silane coupling agents or when their reactivity is limited.

Analysis of the curing curves (Figure 4) revealed that all tested compounds reached a stable torque plateau, indicating the absence of significant material degradation during the measurement. The lack of torque reversion suggests the formation of a stable filler structure within the elastomer matrix. Furthermore, the observed behaviour indicates a satisfactory degree of filler dispersion, which is particularly important in hybrid filler systems.

The absence of torque reversion in all investigated compounds indicates the formation of thermally stable crosslinked networks during vulcanization, which is characteristic of properly optimized sulfur curing systems and agrees with the vulcanization behaviour reported by Akiba and Hashim [14]. The progressive increase in curing torque with increasing carbon black loading is also consistent with the reinforcing mechanism of carbon black described by Fan et al. [15], who attributed this behaviour to increased filler networking, higher compound viscosity, and stronger filler–rubber interactions.

#### 3.1.2. Tensile Strength

The results indicate that the highest mechanical properties among the carbon-black-filled compounds were obtained at a carbon black loading of 20 phr. As the carbon black content increased, the stress values at 100%, 200%, and 300% elongation (SE100, SE200, and SE300), as well as the tensile strength, increased, indicating progressive reinforcement and stiffening of the vulcanizates (Table 2). However, when the carbon black content exceeded 10 phr, a decrease in elongation at break was observed. This behaviour may be attributed to increased stress concentration and an increased crosslink density, which reduced the deformability of the material.

The improvement in stress at fixed elongation and tensile strength with increasing carbon black content agrees well with the established reinforcing role of carbon black in SBR composites described by Fan et al. [15]. The reduction in elongation at break at higher carbon black loadings is likewise consistent with previous studies, in which increasing filler content resulted in higher stiffness and earlier crack initiation due to local stress concentration [15,16].

#### 3.1.3. Hysteresis

The incorporation of carbon black markedly reduced the Mullins effect compared with the silica-filled reference compound (CB0/Sil20), for which the highest Em value (51.8%) was obtained. The lowest Em value (22.6%) was observed for the CB5/Sil15 compound, while the CB1/Sil19 and CB20/Sil0 formulations exhibited comparable values (23.1% and 23.2%, respectively). The CB10/Sil10 sample showed a slightly higher Mullins effect (27.8%) (Table 3). Overall, the carbon black-containing compounds exhibited substantially lower stress softening than the silica-filled reference, although no monotonic relationship between carbon black content and the Mullins effect was observed. These results suggest that partial replacement of silica with carbon black modifies the filler network and filler–rubber interactions, leading to reduced stress softening, whereas further changes in carbon black loading do not produce a consistent trend.

The reduced Mullins effect observed after partial replacement of silica with carbon black suggests that the hybrid filler network becomes less susceptible to strain-induced structural breakdown. Similar behaviour has been reported for filled SBR compounds subjected to cyclic loading, where the fatigue response strongly depends on filler morphology and filler–rubber interactions [3,4,16].

#### 3.1.4. Dynamic Mechanical Analysis

**Figure 5:**
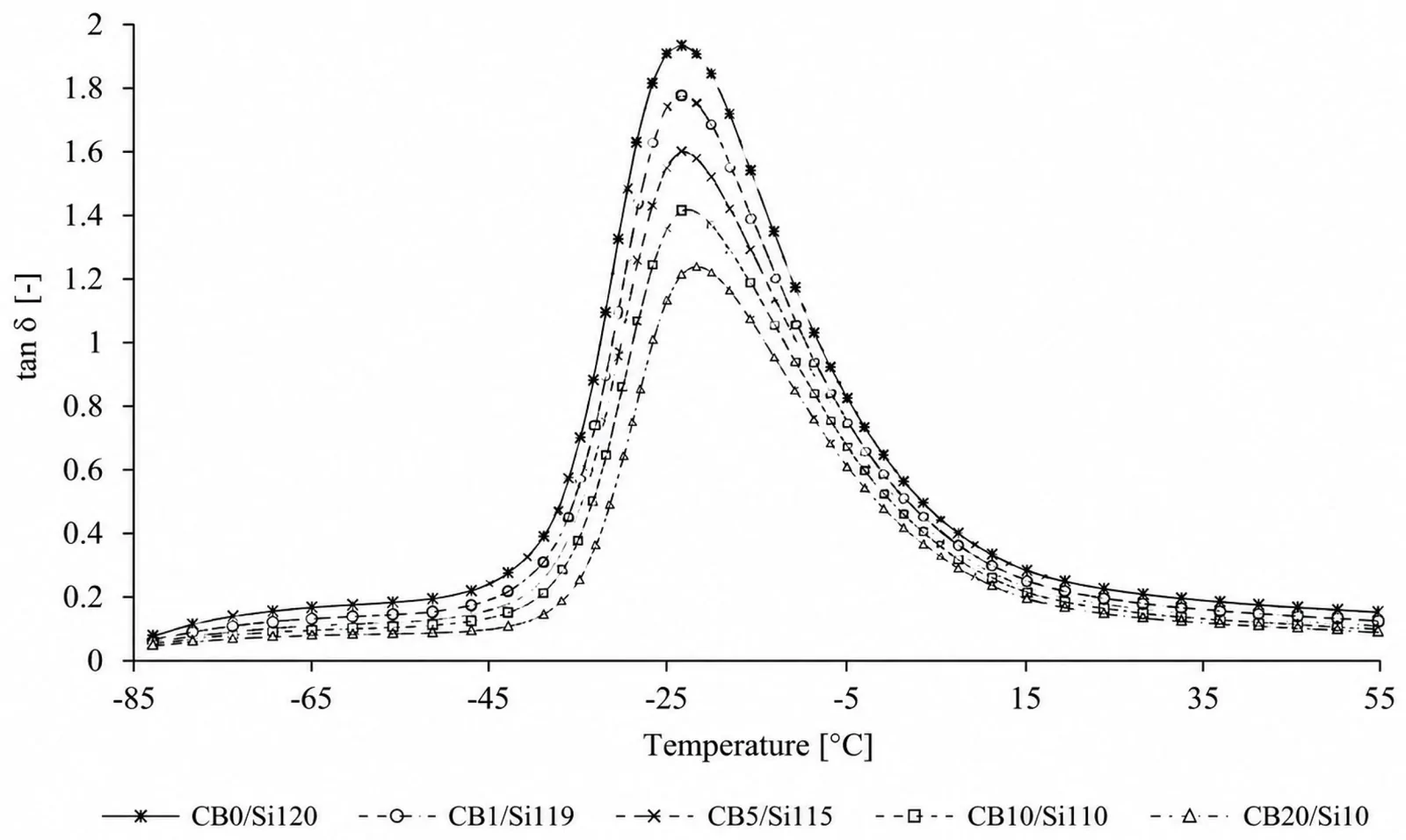
DMA results of carbon black-filled SBR compounds.

When analyzing the DMA results (Figure 5), a clear shift in the glass transition temperature can be observed. The Tg of unfilled styrene-butadiene rubber reaches a value of approximately −45 °C, while the addition of silica and carbon black raises this temperature to approximately −16 to −16.5 °C. Both silica and carbon black limit chain mobility, which can cause an upward shift in Tg. Numerous publications have shown that the presence of fillers can modify local chain segmental motions and thus affect the observed Tg or the width of the tan δ peak in DMA. This effect depends on the type of filler, its surface area, the method of modification (e.g., silanes), the degree of polymer–filler interaction, and the measurement method [4]. In addition, the graph shows a broad peak, indicating a distribution of relaxation times, and high values of the damping factor, tan δ (~1.8–1.9), which may reflect strong viscoelastic losses typical of hybrid compounds or strong filler–rubber interactions.

The decrease in storage modulus with increasing carbon black content indicates progressive weakening of the silica-dominated filler network, whereas the superior modulus of the carbon black-filled compound reflects stronger filler–filler interactions. Similar observations have been reported for hybrid SBR systems, where filler morphology determines the architecture of the reinforcing network [15,17].

#### 3.1.5. Payne Effect

**Figure 6:**
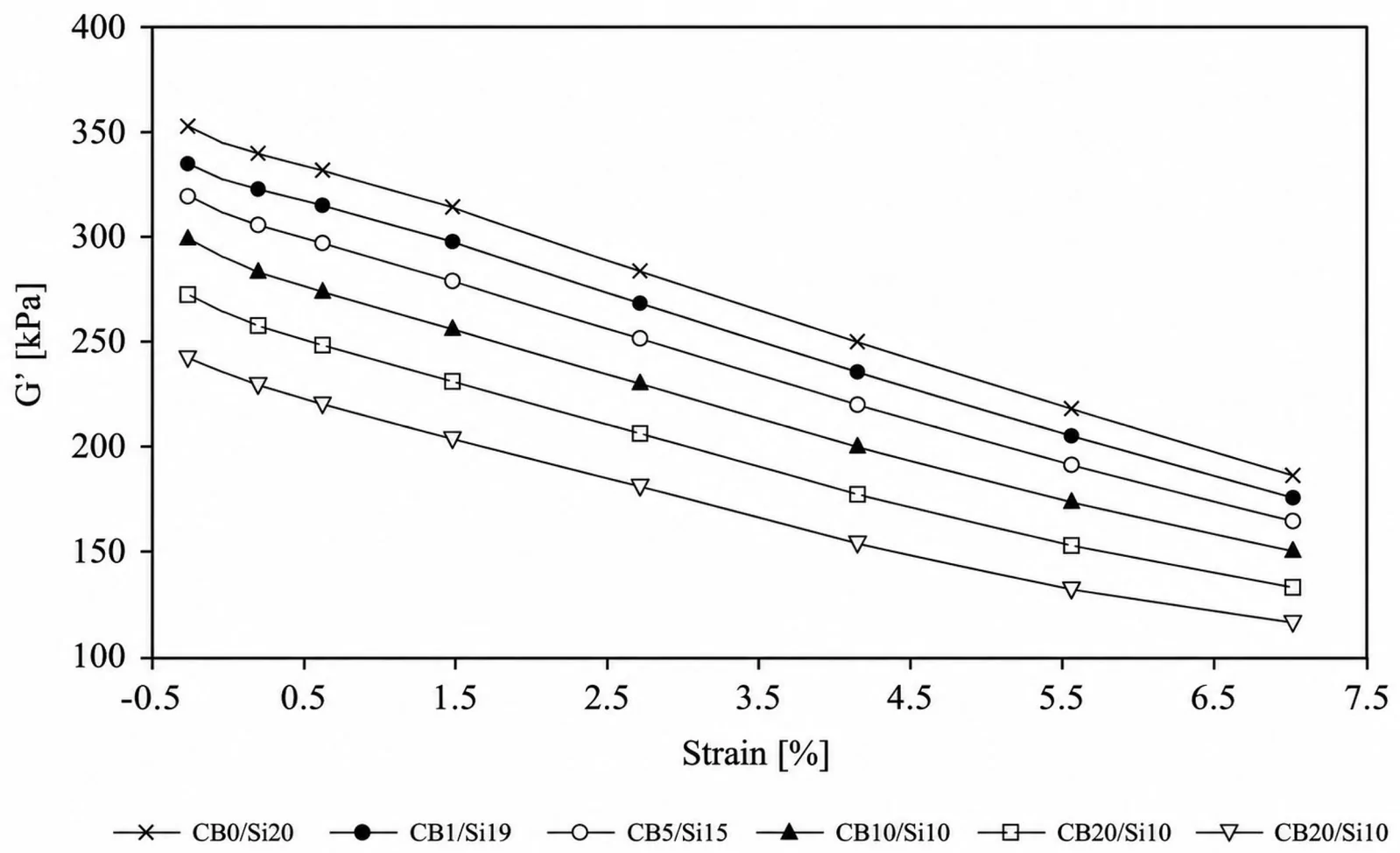
Payne effect results of carbon black-filled SBR compounds.

Figure 6 presents the dependence of the storage modulus (G′) on strain amplitude for S-SBR compounds containing different silica/carbon filler ratios. For all formulations, the storage modulus decreased with increasing strain amplitude, exhibiting the characteristic Payne effect associated with the progressive breakdown of the filler network under dynamic deformation. The highest G′ values were observed for the silica-filled compound (CB0/Sil20), indicating the formation of the strongest filler network. As the carbon filler content increased from 0 to 20 phr, while the silica content was proportionally reduced to maintain a constant total filler loading of 20 phr, the storage modulus gradually decreased over the entire strain range. This behavior suggests a progressive weakening of the filler network with increasing replacement of silica by carbon filler.

The more pronounced Payne effect observed for the silica-rich compounds can be attributed to the stronger filler–filler interactions of silica particles. Owing to their highly polar surface and the presence of silanol groups, silica particles readily form hydrogen-bonded aggregates, resulting in the development of a continuous three-dimensional filler network within the elastomer matrix. Under increasing dynamic strain, these weak physical interactions are progressively disrupted, leading to a reduction in the storage modulus. In contrast, replacing silica with carbon filler decreases the number of silica–silica contacts responsible for the strongest physical interactions, thereby reducing the rigidity and continuity of the filler network.

The hybrid silica/carbon-filled compounds exhibited intermediate G′ values, reflecting the contribution of both fillers to the network structure. However, no synergistic enhancement of the filler network was observed within the investigated composition range. Instead, the gradual decrease in the storage modulus with increasing carbon filler content indicates that the filler network was predominantly governed by the silica phase. Since the Payne effect primarily reflects the strength of filler–filler interactions and the strain-induced breakdown of the filler network, it does not directly quantify the degree of filler dispersion. Nevertheless, the lower G′ values observed for compounds containing higher amounts of carbon filler suggest a reduced extent of filler networking compared with the silica-rich formulations. Confirmation of differences in filler dispersion would require complementary morphological characterization, such as scanning or transmission electron microscopy.

These results demonstrate that, at a constant total filler loading of 20 phr, the type of reinforcing filler has a greater influence on the development and stability of the filler network than the total filler concentration itself, with silica exhibiting a substantially stronger tendency to form a strain-sensitive three-dimensional network than carbon filler.

#### 3.1.6. Temperature Evolution During Cyclic De Mattia Fatigue

**Figure 7:**
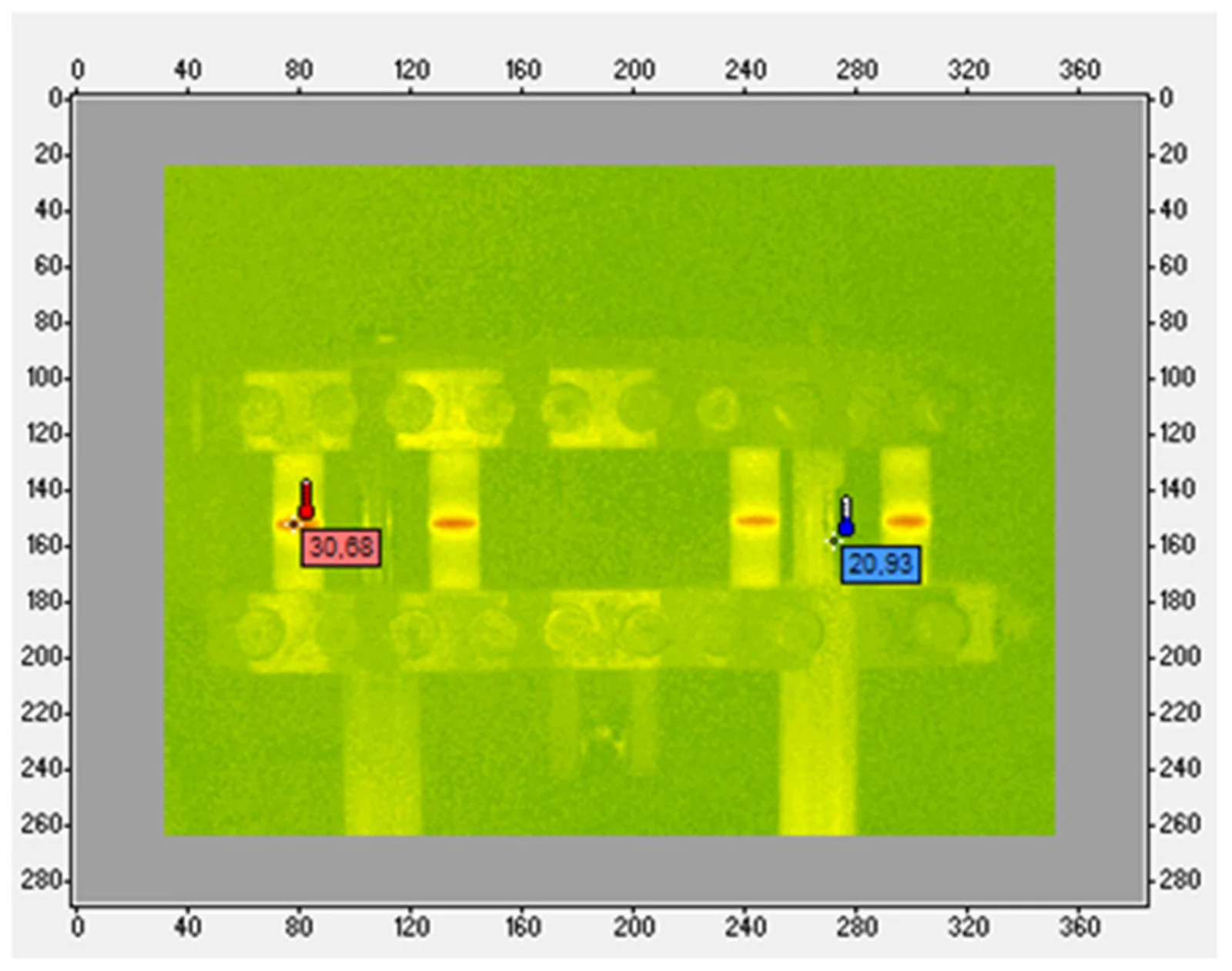
Exemplary thermographic images showing the temperature distribution in the compounds with varying carbon black content; after 2000 De Mattia bending cycles.

The temperature evolution during cyclic De Mattia flexing is summarized in Table 4, while an exemplary thermographic image is presented in Figure 7. For all investigated compounds, the temperature increased with the number of loading cycles, indicating progressive heat accumulation during cyclic deformation. However, the magnitude of the temperature rise depended strongly on the carbon black/silica ratio.

Among the carbon black–silica hybrid systems, the highest temperature increase after 10,000 cycles was observed for the CB5/Sil15 compound (4.5 °C), followed by CB20/Sil0 (3.9 °C) and CB10/Sil10 (3.4 °C). In contrast, the CB1/Sil19 compound exhibited the lowest temperature rise (0.7 °C), whereas the silica-filled reference (CB0/Sil20) reached an intermediate value of 2.4 °C.

The results indicate that heat build-up during cyclic deformation is governed by the composition of the hybrid filler system rather than by the carbon black content alone. No monotonic relationship between carbon black loading and temperature rise was observed. Instead, the maximum temperature increase occurred at an intermediate carbon black loading of 5 phr, while a further increase to 20 phr did not lead to additional heat generation. Since the temperature rise under cyclic loading is directly associated with hysteresis losses and energy dissipation, these findings suggest that the carbon black/silica ratio strongly influences the viscoelastic response of the rubber compounds and their ability to dissipate mechanical energy as heat.

#### 3.1.7. Thermal Conductivity

The thermal conductivity of the investigated SBR compounds was determined at 10, 20, and 40 °C, and the results are presented in Table 5. For all formulations, a slight increase in thermal conductivity with increasing measurement temperature was observed, which is characteristic of elastomeric materials. However, the differences between the investigated compounds remained relatively small over the entire temperature range.

Among the carbon-black-filled compounds, the lowest thermal conductivity was recorded for the CB1/Sil19 formulation, whereas the CB5/Sil15 compound exhibited the highest values. Further increasing the carbon black content to 10 and 20 phr did not result in a significant improvement in thermal conductivity, with the CB10/Sil10 and CB20/Sil0 compounds showing values comparable to those of CB5/Sil15. These results indicate that thermal transport in the investigated hybrid silica/carbon black systems is not governed solely by carbon black loading. Instead, the efficiency of heat conduction appears to depend on the morphology and dispersion of the filler network, as well as the interactions between silica and carbon black within the rubber matrix.

Although the measured differences in thermal conductivity were relatively small, they provide valuable insight when considered together with the thermographic analysis performed during cyclic De Mattia flexing. The temperature evolution under cyclic loading could not be explained solely by the thermal conductivity of the materials. Instead, the observed self-heating behaviour may reflect the combined effects of heat generation through viscoelastic energy dissipation and heat transfer within the filler network. Consequently, the thermal response of the investigated compounds may be influenced by both their thermal transport properties and their hysteretic behaviour under repeated deformation.

#### 3.1.8. Crosslink Density

**Figure 8:**
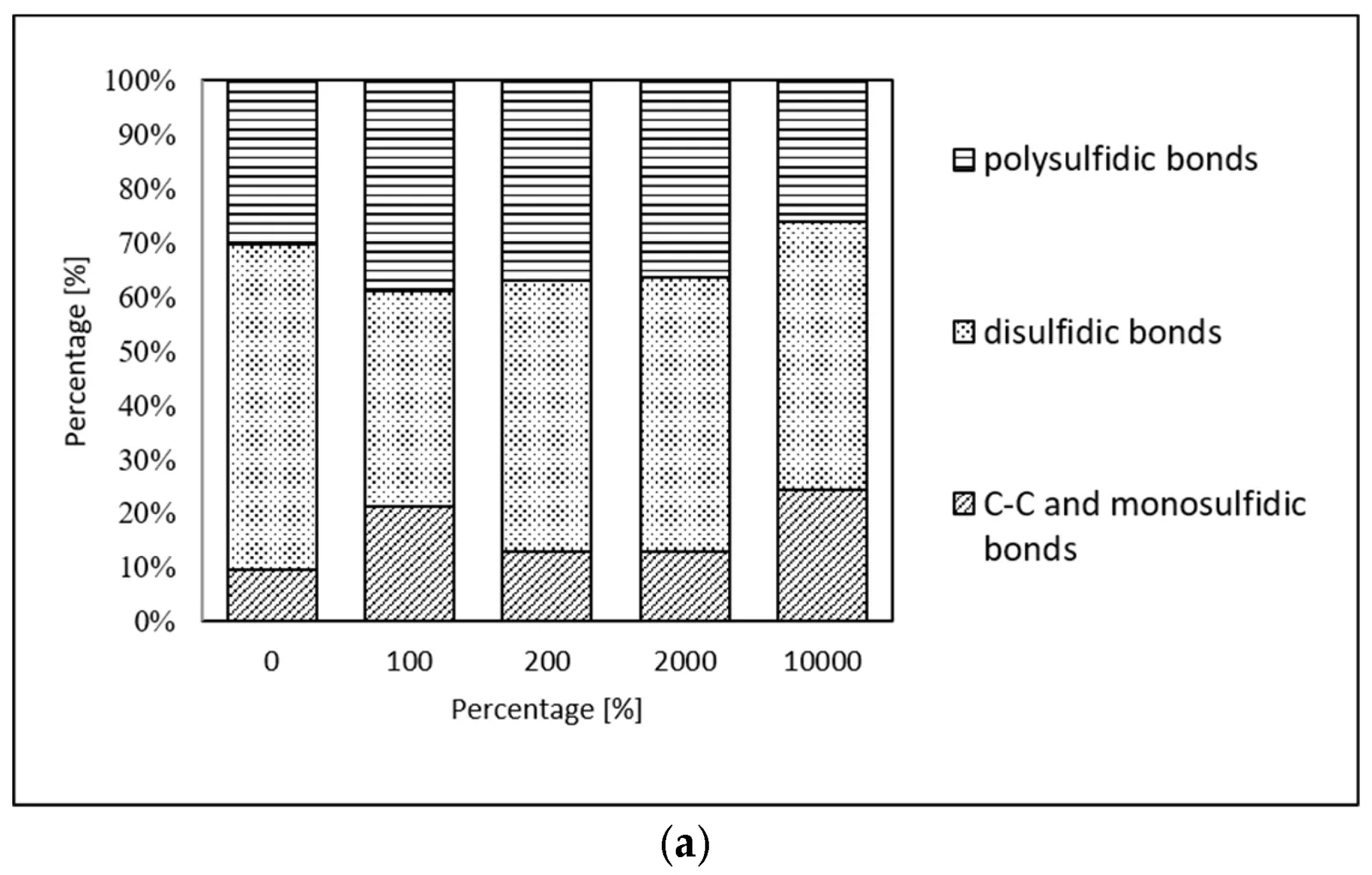

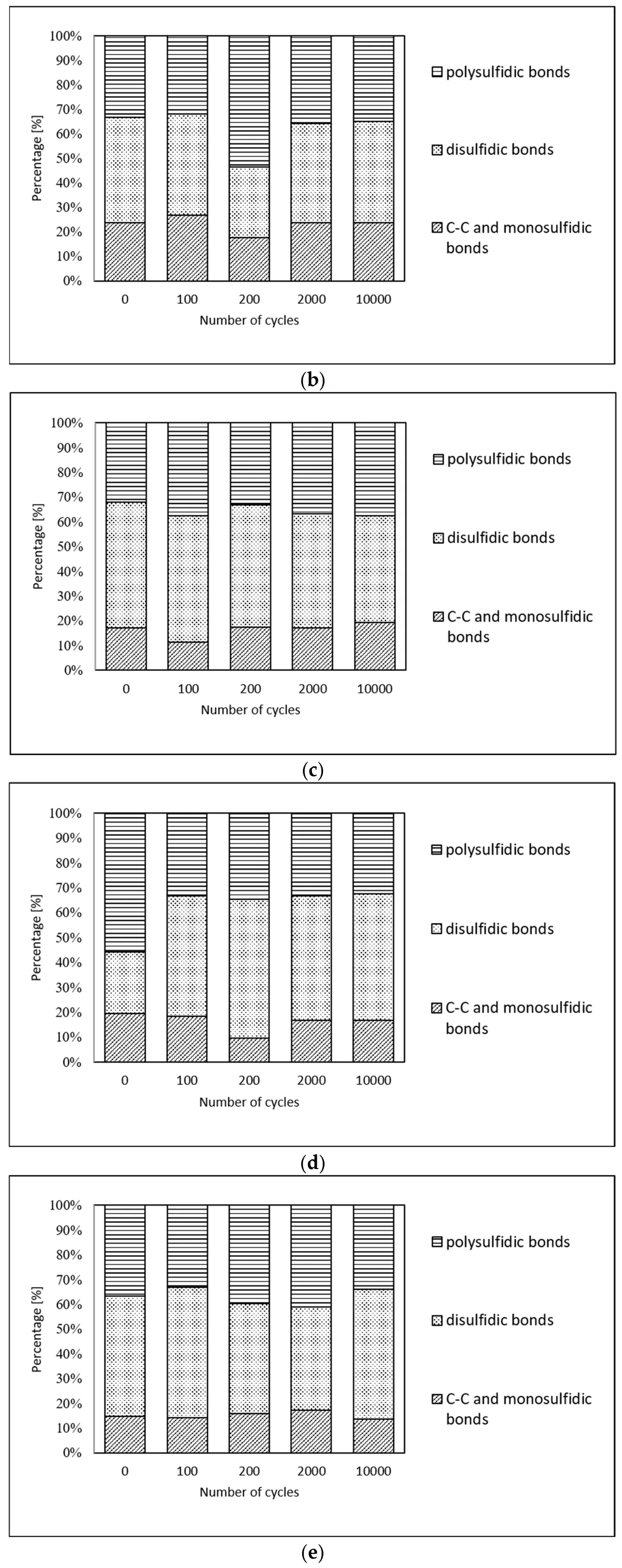
Crosslink density of carbon black-filled SBR compounds. ((**a**)—CB0/Sil20, (**b**)—CB1/Sil19, (**c**)—CB5/Sil15, (**d**)—CB10/Sil10, (**e**)—CB20/Sil0).

The results indicate that the crosslink density (Figure 8) increased with increasing carbon black content. During dynamic ageing, an initial decrease in crosslink density was observed after approximately 100 loading cycles, followed by a subsequent increase and a slight decrease at later stages of the ageing process. The initial reduction may be associated with possible rearrangement of sulfur crosslinks, including the conversion of disulfidic bonds into mono- and polysulfidic structures. At approximately 10,000 cycles, an increase in the concentration of monosulfidic and C-C bonds was observed, which may suggest the occurrence of additional crosslinking reactions.

The magnitude of these changes decreased with increasing carbon black content. This behaviour may be related to the higher stiffness of the material, which limits molecular mobility and, consequently, the extent of further possible network reorganization. Moreover, the relatively small changes in the concentration of monosulfidic and C-C bonds at higher carbon black loadings may indicate a possible free-radical scavenging effect, which may reduce the formation of new bonds between polymer chains. This phenomenon was not observed for the reference compound containing only silica.

#### 3.1.9. Microhardness

**Figure 9:**
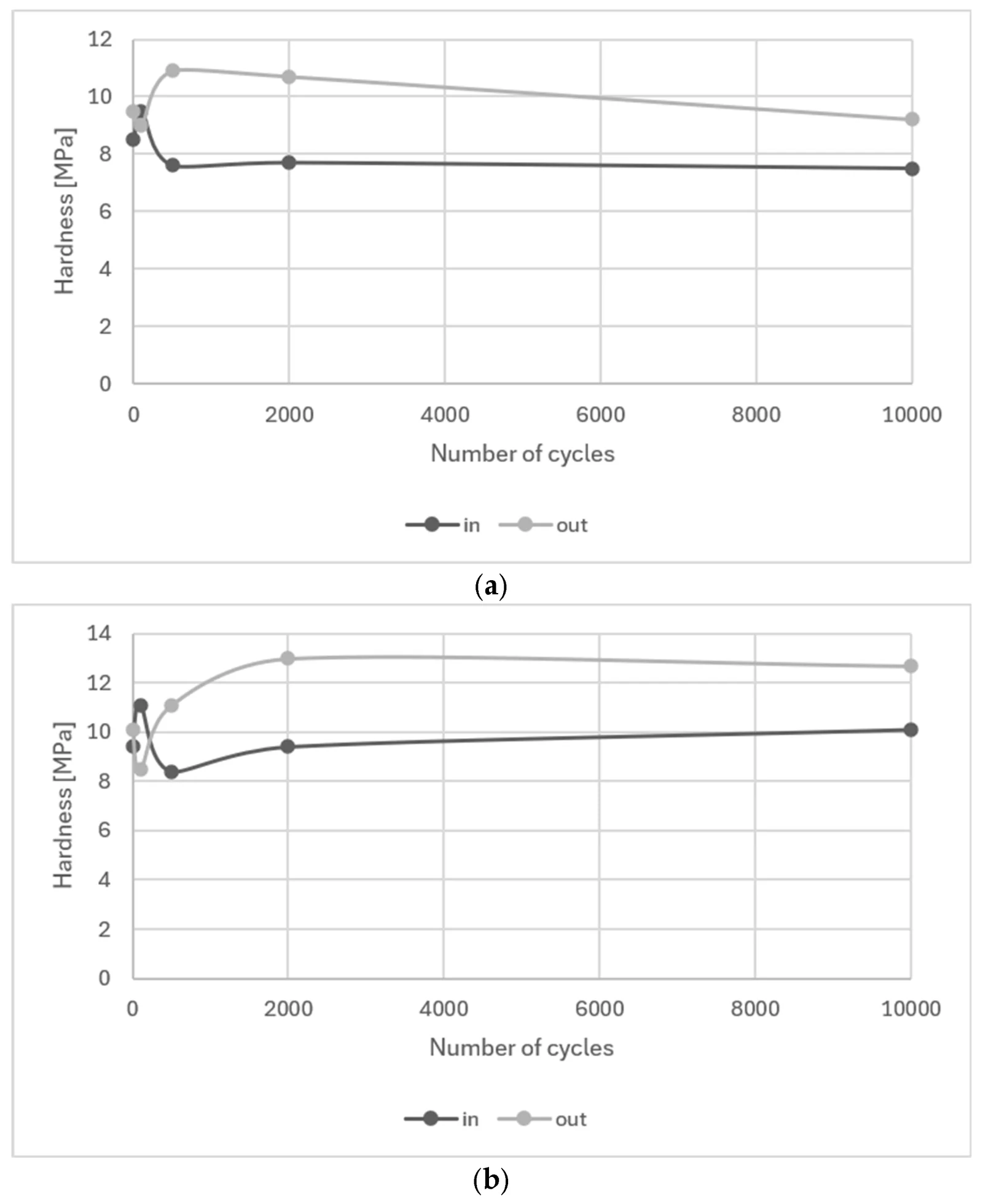

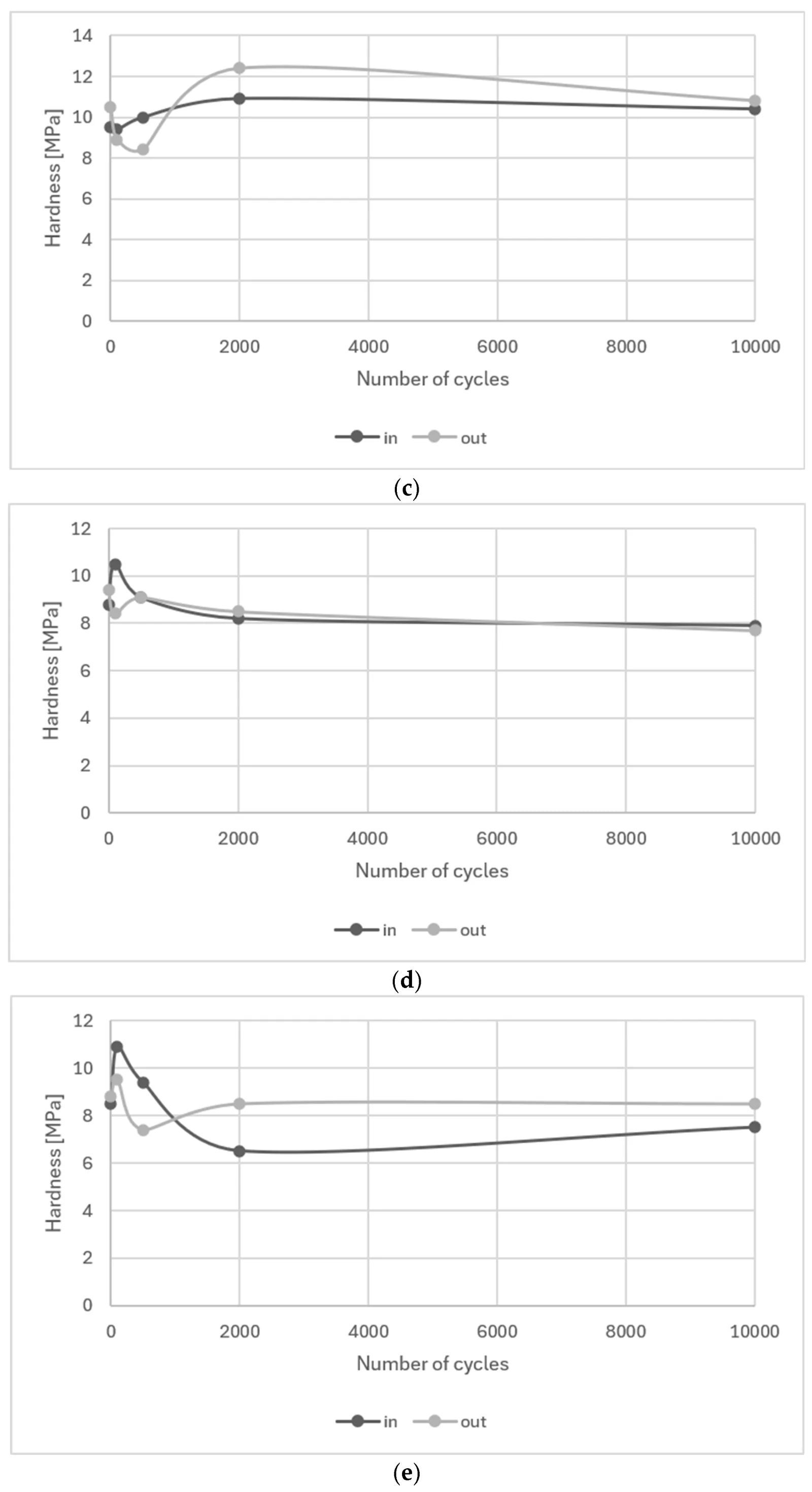
Microhardness of carbon black-filled SBR compounds. ((**a**)—CB0/Sil20, (**b**)—CB1/Sil19, (**c**)—CB5/Sil15, (**d**)—CB10/Sil10, (**e**)—CB20/Sil0).

For the silica-filled reference compound, a slight increase in hardness was observed in the initial stage of dynamic ageing, followed by a decrease and subsequent stabilization. A similar trend was observed at the surface, where a slight decrease in hardness was followed by an increase and a further reversal of the trend (Figure 9). These changes may be associated with the competing effects of polymer chain scission and possible network reorganization occurring during cyclic loading. Chain degradation may lead to a reduction in hardness, whereas partial recombination and the formation of new crosslinks may contribute to its recovery.

The addition of 1 phr of carbon black did not result in significant changes compared with the silica-filled reference compound. In contrast, the compounds containing 5 and 10 phr of carbon black showed no noticeable deterioration in hardness within the bulk of the material throughout the ageing process. In particular, the compound containing 10 phr of carbon black exhibited the most stable surface hardness (Figure 9). This behaviour may be related to several factors, including possible differences in heat transfer and thermal distribution during cyclic deformation.

Further increasing the carbon black content to 20 phr resulted in a reduction in hardness both at the surface and in the bulk of the material (Figure 9). This behaviour may be attributed to excessive stiffening of the elastomer network, which can increase stress concentration during cyclic loading and promote structural damage.

### 3.2. Comparison of the Effect of Various Carbon Fillers Added in an Amount of 1 phr

In this section, the results are compared for compounds containing an identical amount (1 phr) of different carbon fillers. Therefore, the discussion focuses on the influence of filler type under otherwise identical formulation conditions, rather than on identifying the optimum loading of each filler.

#### 3.2.1. Curing Kinetics

**Figure 10:**
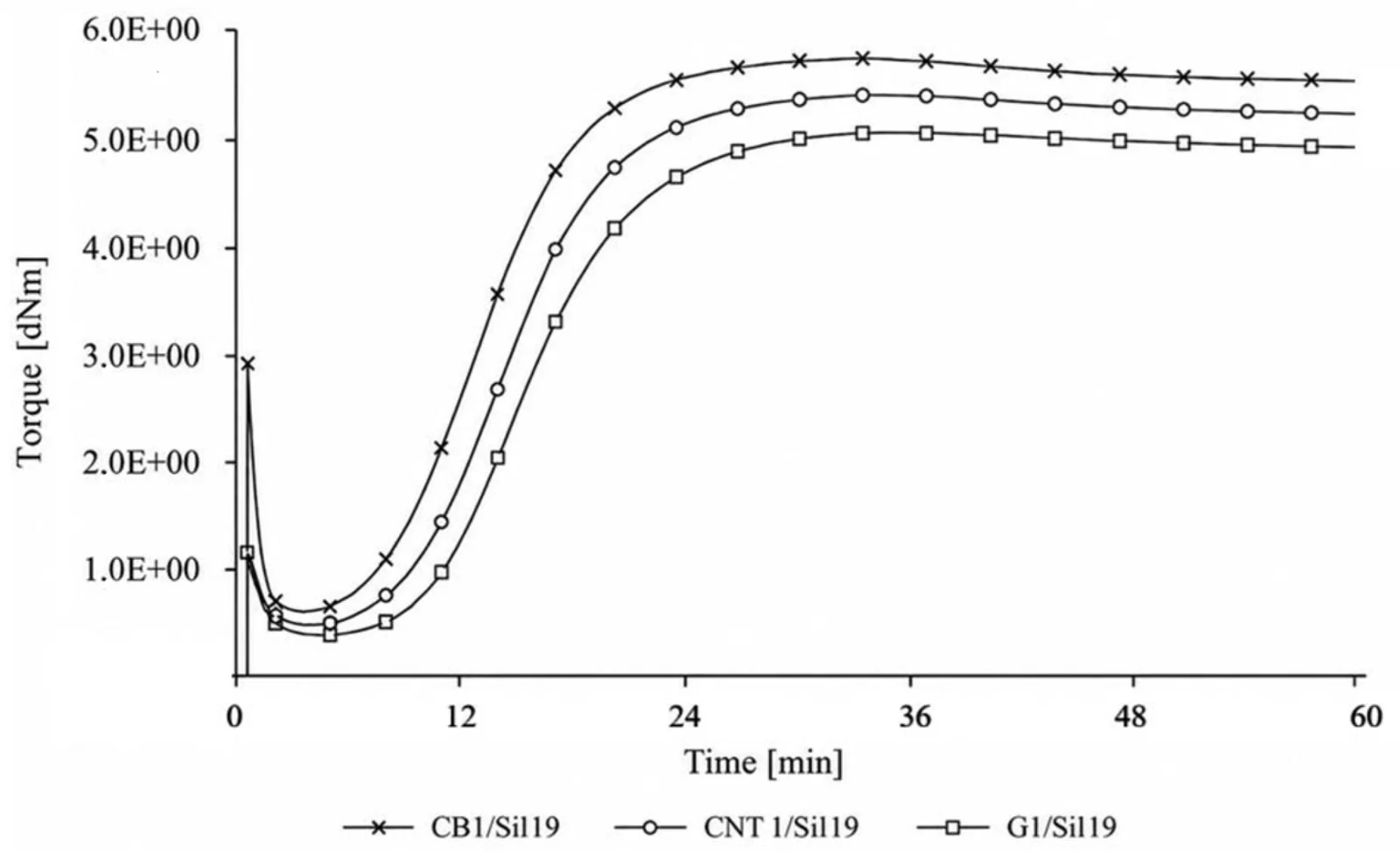
Curing kinetics of SBR compounds filled with different carbon fillers.

The torque-time curves revealed a clear hierarchy of plateau values: G1/Sil19 > CNT1/Sil19 > CB1/Sil19 (Figure 10). Among the investigated fillers, graphene produced the highest plateau torque, followed by carbon nanotubes (CNTs), while carbon black generated the lowest value. This behaviour is likely related to differences in filler morphology and dimensionality. The two-dimensional structure of graphene provides an extensive interfacial contact area with the rubber matrix, whereas the one-dimensional geometry of CNTs facilitates the formation of interconnected filler networks. Consequently, at the same filler loading, carbon black forms the least developed filler network.

All investigated hybrid systems reached a stable torque plateau without any noticeable decrease during the measurement (Figure 10). This behaviour indicates good curing stability and the absence of significant thermomechanical degradation under the applied test conditions. Furthermore, the results suggest that the incorporation of carbon nanofillers into the silica-filled compounds does not adversely affect the stability of the filler network during curing.

The highest curing torque observed for graphene-filled compounds further confirms the superior networking ability of high-aspect-ratio nanofillers, in agreement with the reviews by Kausar [18] and Ali et al. [19], which emphasize the exceptionally large specific surface area of graphene and its ability to establish an interconnected reinforcing network even at low concentrations.

#### 3.2.2. Tensile Strength

The incorporation of carbon fillers resulted in higher stress values at a given level of elongation. A comparison of the effects of the individual fillers on the mechanical properties of the vulcanizates showed that the graphene-filled compound exhibited the highest tensile strength and the greatest elongation at break (Table 6). These findings indicate that graphene provided the most effective reinforcement, resulting in improved stiffness while maintaining a high capacity for deformation.

Graphene exhibited the highest reinforcing efficiency among the investigated nanofillers, simultaneously improving tensile strength and maintaining high elongation at break. Similar behaviour has been reported for graphene-reinforced elastomers, where the large aspect ratio and extensive polymer–graphene interface promote efficient stress transfer while preserving deformability [18,19,20].

#### 3.2.3. Hysteresis

A comparative analysis revealed that the Mullins effect followed the trend CNT1/Sil19 > G1/Sil19 > CB1/Sil19 (Table 7). Despite the differences in filler morphology and structure, the incorporation of carbon nanofillers did not result in a reduction in this phenomenon. Compared with the compound containing the same amount of carbon black, the nanofiller-filled vulcanizates exhibited higher Mullins effect values, indicating more pronounced stress-induced structural changes during cyclic deformation.

The larger stress softening exhibited by graphene- and CNT-filled compounds indicates stronger polymer–nanofiller interactions and may indicate greater possible network reorganization during repeated deformation, which is consistent with the fatigue behaviour reported for graphene- and CNT-reinforced SBR composites [21,22].

#### 3.2.4. Dynamic Mechanical Analysis

**Figure 11:**
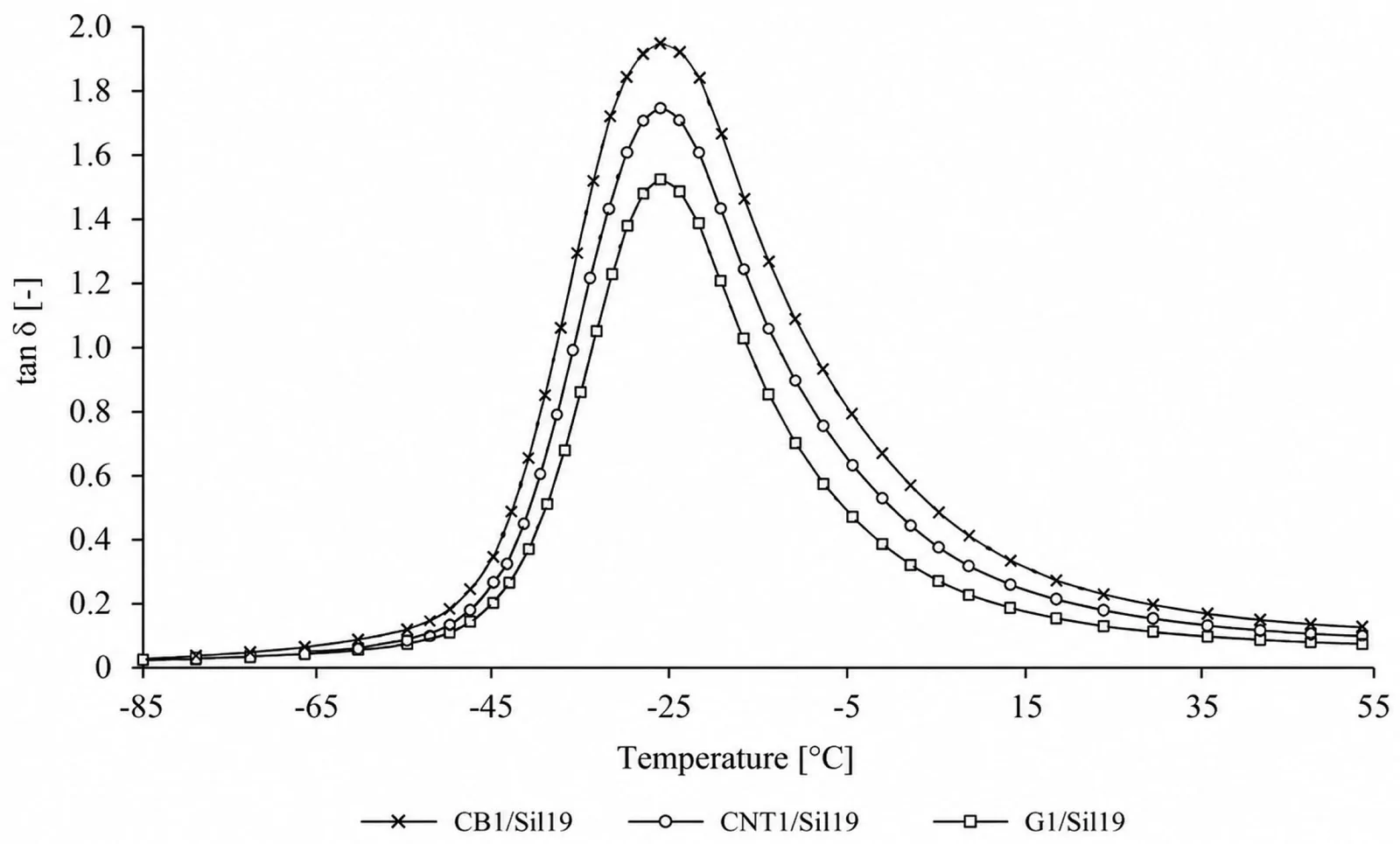
DMA results of SBR compounds filled with different carbon fillers.

For the compounds containing 1 phr of carbon black or graphene, higher tan δ values were observed, indicating greater energy dissipation during dynamic deformation (Figure 11).

In contrast, the compound filled with carbon nanotubes exhibited a lower tan δ value, suggesting a more elastic response of the material. This behaviour may be attributed to several factors, including the possible formation of a more effective and interconnected filler network by the nanotubes, which may limit energy dissipation and suppress viscoelastic chain motions in the vicinity of the glass transition temperature (Tg).

In contrast to carbon black, graphene and CNTs produced mechanically more compliant networks that remained more strain-tolerant during deformation, consistent with previous reports demonstrating that carbon nanofillers improve stress transfer primarily through strong polymer–filler interactions rather than by forming highly rigid filler aggregates [18,21].

#### 3.2.5. Payne Effect

**Figure 12:**
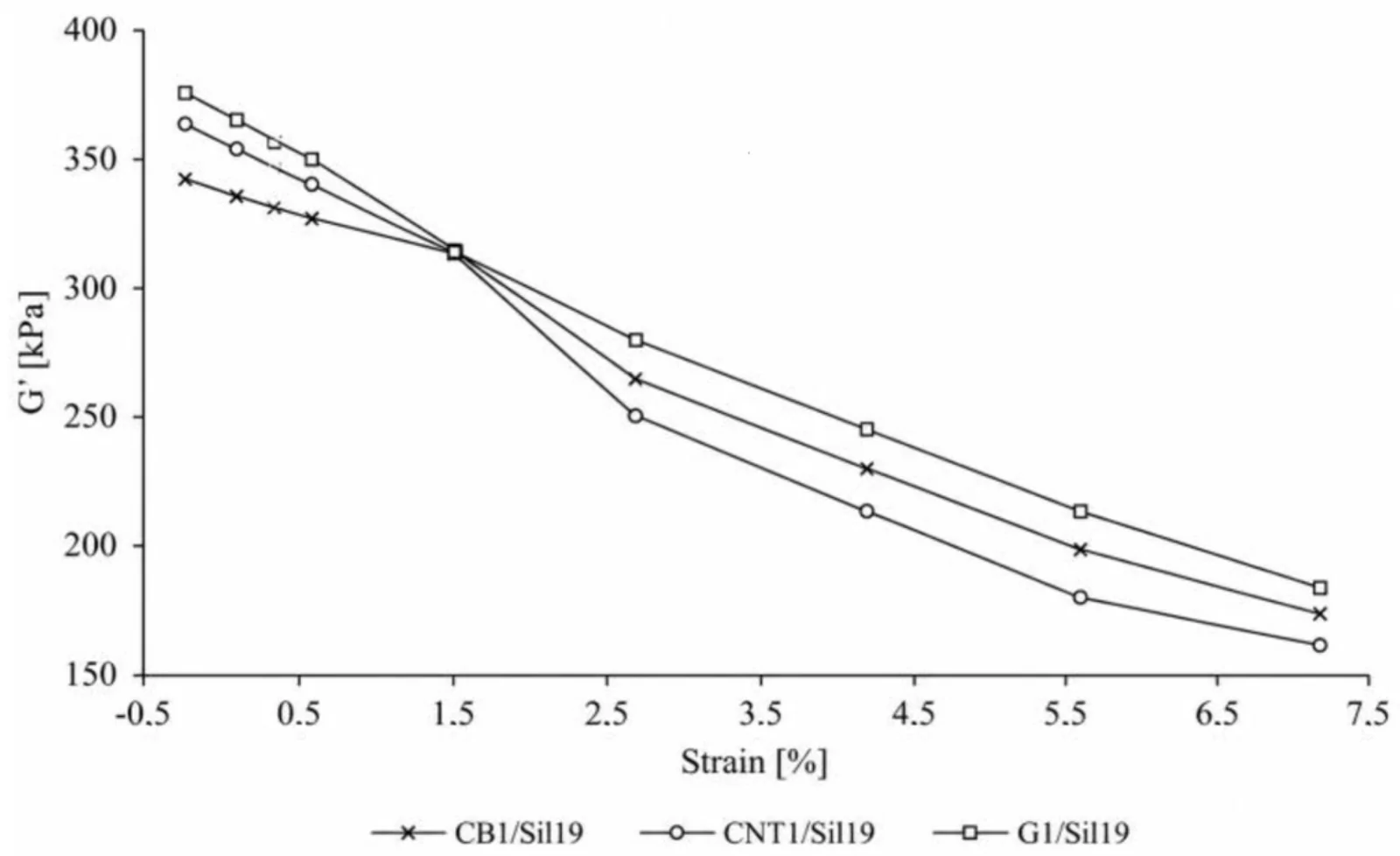
Payne effect results of SBR compounds filled with different carbon-based fillers at 1 phr loading.

All compounds exhibited the characteristic decrease in storage modulus (G′) with increasing strain amplitude, reflecting the progressive breakdown of the filler network under dynamic deformation. The differences between the investigated systems were relatively small; however, the carbon-black-filled compound (CB1/Sil19) exhibited the highest G′ values over the entire strain range, indicating the strongest filler network and the most pronounced filler–filler interactions. In contrast, the CNT1/Sil19 and G1/Sil19 compounds showed slightly lower storage modulus values, suggesting a less developed filler network (Figure 12).

Since the silica content and the total filler loading were kept constant (19 and 20 phr, respectively), the observed differences can be attributed primarily to the type of carbon filler introduced into the hybrid filler system. Carbon black, owing to its aggregate morphology and high specific surface area, promotes the formation of a physically interconnected filler network in combination with silica. Conversely, the incorporation of carbon nanotubes or graphene modifies the architecture of the hybrid filler structure, resulting in a lower extent of filler–filler interactions, as evidenced by the reduced storage modulus.

The decrease in G′ with increasing strain amplitude confirms that the filler network in all compounds is disrupted under dynamic loading, which is characteristic of the Payne effect. The slightly lower Payne effect observed for the CNT- and graphene-containing compounds suggests that these fillers contribute to a network that is less susceptible to strain-induced breakdown than that formed in the carbon-black-containing compound. This behaviour may be related to differences in the morphology and spatial arrangement of the carbon fillers within the silica-reinforced S-SBR matrix.

It should be noted that the Payne effect primarily reflects the strength of filler–filler interactions and the stability of the filler network rather than the degree of filler dispersion. Therefore, although the results indicate differences in the filler network architecture resulting from the type of carbon filler, direct conclusions regarding filler dispersion require complementary morphological characterization, such as scanning or transmission electron microscopy.

#### 3.2.6. Temperature Evolution During Cyclic De Mattia Fatigue

**Figure 13:**
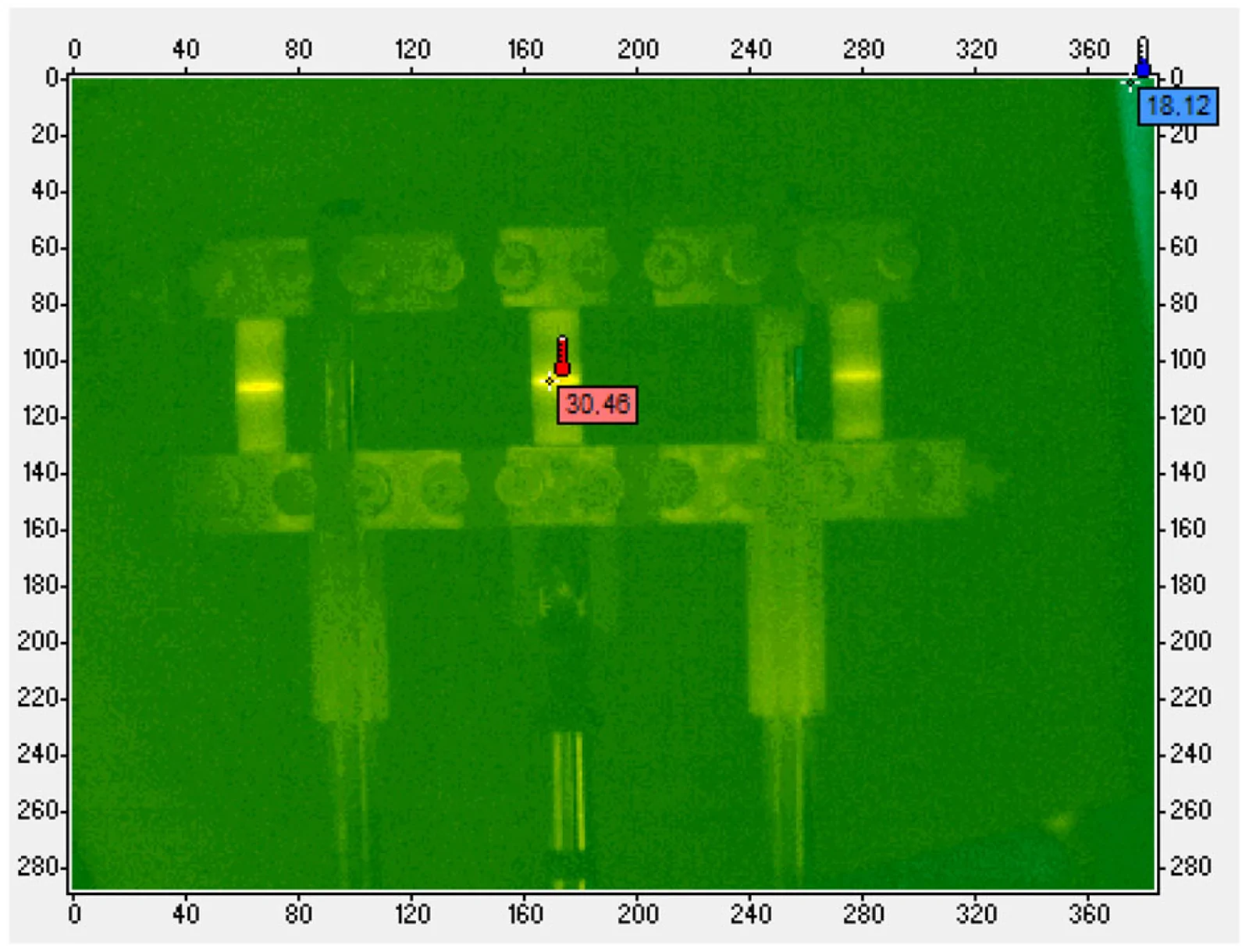
Exemplary thermographic images showing the temperature distribution in the compounds containing carbon nanofillers (CNT and graphene) after 2000 De Mattia bending cycles.

The influence of carbon nanofillers was evaluated by comparing compounds containing 1 phr of graphene (G1/Sil19) or carbon nanotubes (CNT1/Sil19) with the CB1/Sil19 formulation. The temperature evolution during cyclic loading is summarized in Table 8, while an exemplary thermographic image is presented in Figure 13. The incorporation of different nanofillers produced markedly different thermal responses during cyclic loading. The graphene-containing compound reached a temperature increase of only 1.5 °C after 10,000 cycles, whereas the CNT-filled compound exhibited a considerably higher temperature rise of 3.4 °C.

These results demonstrate that heat build-up is governed not only by thermal conductivity but also by hysteretic energy dissipation. Although graphene increased the thermal conductivity of the composites, it simultaneously resulted in substantially lower self-heating during cyclic deformation, suggesting reduced mechanical energy losses and/or more efficient heat dissipation. Consequently, the type of carbon nanofiller modifies the viscoelastic response of the rubber compounds during repeated deformation and may influence their resistance to thermo-mechanical ageing.

Similar observations were reported by Xu et al. [22], who demonstrated that graphene oxide and carbon nanotubes improve the fatigue performance of silica/SBR composites by limiting heat accumulation during cyclic deformation. Moreover, the exceptionally high thermal conductivity of graphene reported in the literature may further enhance heat dissipation from the material [18,19].

#### 3.2.7. Thermal Conductivity

The thermal conductivity measurements (Table 9) showed that the graphene-filled compound exhibited the highest thermal conductivity over the entire investigated temperature range, followed by the CNT-filled and carbon-black-filled formulations. This behaviour can be attributed to the intrinsically high thermal conductivity of graphene and its ability to establish efficient heat-transfer pathways within the polymer matrix. Carbon nanotubes also enhanced thermal conductivity compared with carbon black, owing to their high aspect ratio and the formation of conductive networks. In contrast, the carbon-black-filled compound exhibited the lowest thermal conductivity, reflecting the lower efficiency of particulate fillers in facilitating heat transport.

Although the differences in thermal conductivity were relatively small, they are consistent with the thermographic observations obtained during cyclic De Mattia flexing. The graphene-filled compound, which exhibited the highest thermal conductivity, also showed a relatively low temperature rise under cyclic loading, which may suggest more efficient heat dissipation. However, the CNT-filled compound displayed a considerably higher temperature increase despite its improved thermal conductivity. This indicates that the temperature evolution during cyclic deformation is governed not only by the ability of the material to conduct heat but also by the amount of heat generated through viscoelastic energy dissipation. Consequently, both the thermal transport properties and the hysteretic behaviour of the filler network may contribute to the overall self-heating response of the investigated rubber compounds.

#### 3.2.8. Crosslinking Density

**Figure 14:**
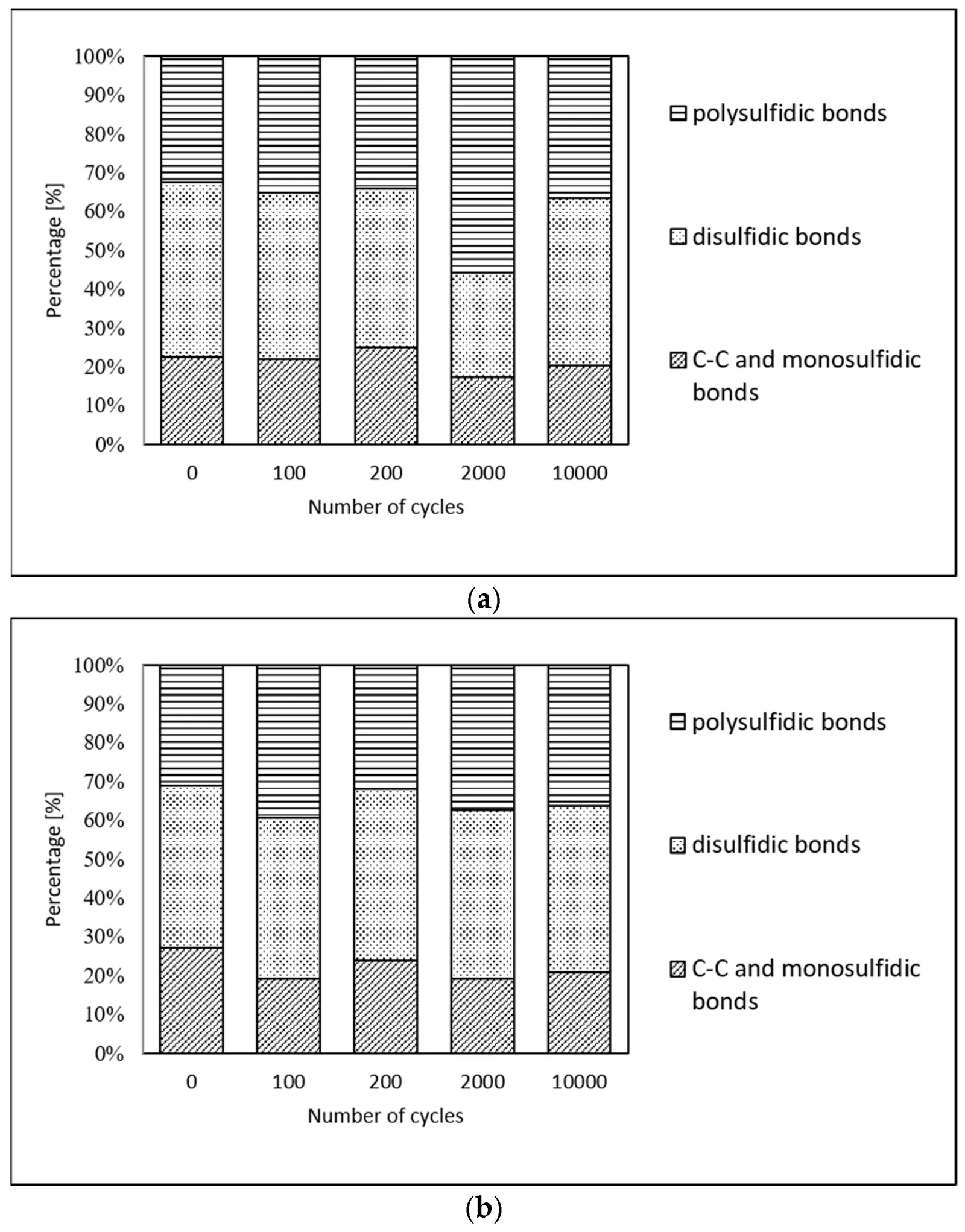
Crosslinking density of SBR compounds filled with different carbon fillers. ((**a**)—G1/Sil19, (**b**)—CNT1/Sil19).

The incorporation of graphene did not provide the same degree of stabilization during the initial stage of dynamic ageing as observed for carbon black. The results indicate an increase in the proportion of monosulfidic and C-C crosslinks, which may suggest ongoing possible network reorganization and additional crosslinking reactions (Figure 14). In contrast, the presence of carbon nanotubes may have contributed to a marked stabilization of the crosslink structure throughout the ageing process.

#### 3.2.9. Microhardness

**Figure 15:**
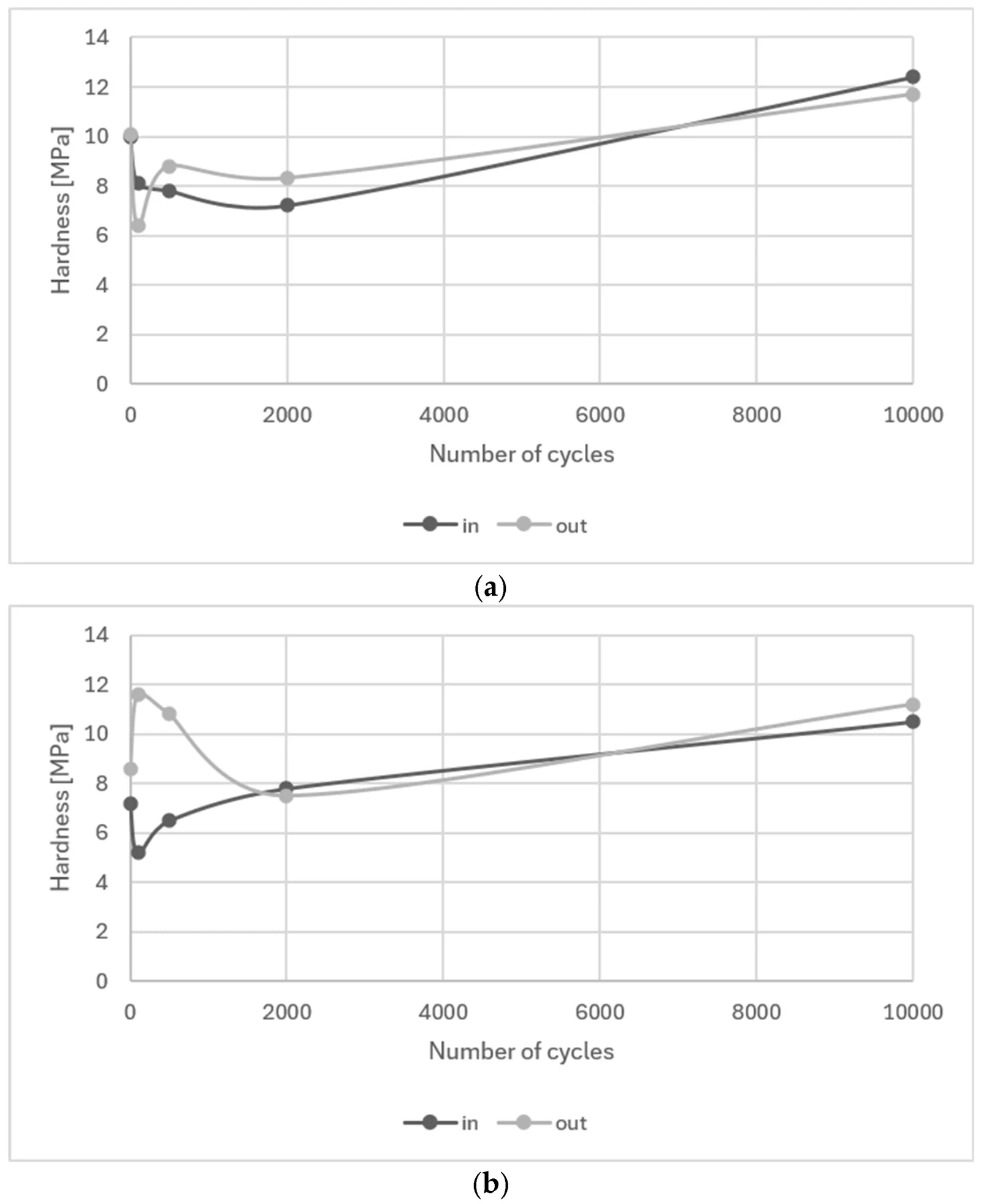
Microhardness of SBR compounds filled with different carbon fillers. ((**a**)—G1/Sil19, (**b**)—CNT1/Sil19).

For the compounds filled with graphene and carbon nanotubes, a decrease in hardness was observed within the bulk of the material during the initial stages of dynamic ageing. Both materials remained relatively stable up to approximately 2000 loading cycles. Beyond this point, however, an increase in hardness was detected. Interestingly, this behaviour was not accompanied by a corresponding increase in crosslink density, suggesting that mechanisms other than possible network reorganization may contribute to the observed hardening (Figure 15). Possible explanations include changes in filler–rubber interactions, local structural changes within the elastomer matrix, or the formation of regions with restricted chain mobility. As the origin of this phenomenon remains unclear, further investigation is required.

### 3.3. Analysis of Volatile Compounds Released After Aging

Analysis of the GC-IMS spectra (Table 10) revealed a relationship between compound composition and the profile of volatile compounds released during dynamic ageing. Cyclic loading promotes bond scission, possible network reorganization, and polymer chain degradation, leading to the formation of volatile species that can be detected by GC-IMS. Changes in carbon black content, as well as the incorporation of carbon nanofillers, influenced the shape and intensity of the recorded signals, indicating differences in the degradation behaviour of the investigated compounds.

In the GC-IMS spectra, the x-axis represents the drift time (ms), corresponding to ion separation within the IMS drift tube, whereas the y-axis represents the chromatographic retention time (s). The intense vertical red line corresponds to the Reactive Ion Peak (RIP), which serves as a reference signal for the detected analytes. Compounds appearing to the right of the RIP (indicated by the arrow) exhibit lower ion mobility than the reactant ions. The position and intensity of these signals provide qualitative and semi-quantitative information about the volatile organic compound (VOC) profile generated during ageing.

Based on a literature survey, several potential markers of rubber degradation were selected for analysis [23], including benzene, toluene, m-xylene, o-xylene, p-xylene, tert-butylamine, and benzothiazole. Among the compounds investigated, benzothiazole and m-xylene were detected in the aged rubber specimens. Their presence confirms that dynamic ageing induced chemical changes within the elastomer network, resulting in the formation and release of volatile degradation products.

**Figure 16:**
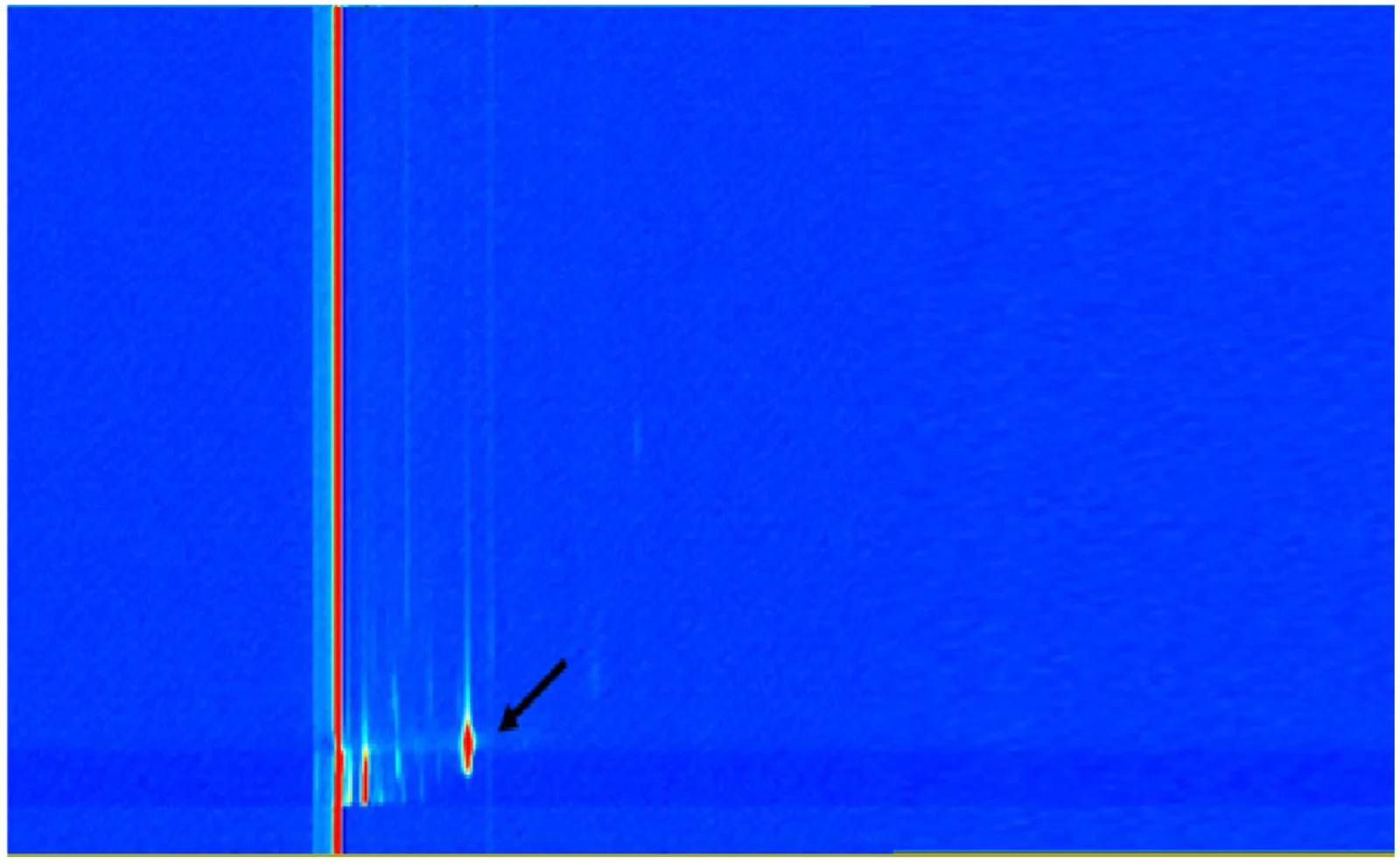
GC-IMS spectra aged samples. The arrow shows repeating signal.

To further evaluate the characteristic signal indicated in Figure 16, semi-quantitative GC-IMS parameters, including the maximum signal height, peak volume, peak area, and ion current, were analysed. All four parameters exhibited the same trend, confirming that the observed differences resulted primarily from changes in the abundance of the detected volatile compound rather than from shifts in its chromatographic retention time or ion mobility. The highest values of all signal descriptors were observed for the silica-filled reference compound (CB0/Sil20) and for formulations containing low amounts of carbon black (CB1/Sil19-CB10/Sil10), with the strongest response recorded for the CB5/Sil15 formulation. These results indicate the highest emission of volatile degradation products during dynamic ageing in compounds with a high silica content or low carbon black loading. In contrast, increasing the carbon black content to 20 phr led to a marked reduction in signal intensity, peak volume, peak area, and ion current. The lowest values were obtained for the compounds containing carbon nanofillers (CNT1/Sil19 and G1/Sil19), demonstrating a further decrease in the abundance of the detected volatile species. Overall, the results suggest that increasing the proportion of carbon fillers, particularly carbon nanofillers, suppresses the formation and/or release of volatile degradation products during cyclic ageing, indicating enhanced resistance of the elastomer network to thermo-mechanical degradation.

The GC-IMS results complement the mechanical observations by providing direct evidence of thermo-mechanical degradation. The formation of benzothiazole and aromatic volatile compounds confirms that repeated cyclic loading induces chemical degradation of the elastomer network, in agreement with recent reviews of elastomer degradation mechanisms [24]. The progressive reduction in volatile degradation products with increasing carbon black loading may be consistent with the protective role of reinforcing fillers against thermo-oxidative degradation [15]. The lowest abundance of degradation products observed for graphene- and CNT-filled compounds is also consistent with reports describing the possible radical scavenging activity of carbon nanotubes [17] and the antioxidant behaviour of graphene-based materials [25], both of which may contribute to improved resistance to oxidation-induced degradation.

## 4. Discussion

This work systematically investigated the influence of hybrid silica/carbon filler systems on the curing behaviour, mechanical performance, dynamic properties, thermal transport, thermo-mechanical ageing, and degradation behaviour of S-SBR vulcanizates. Two complementary approaches were employed: (i) the progressive replacement of silica with carbon black while maintaining a constant total filler loading of 20 phr and (ii) the substitution of 1 phr silica with carbon fillers of different morphology, namely carbon black, carbon nanotubes (CNTs), and graphene. Together, these approaches demonstrate that both the concentration and, more importantly, the morphology and dimensionality of carbon fillers govern the development of the reinforcing network and the long-term performance of hybrid elastomer composites.

All investigated compounds exhibited stable curing behaviour without torque reversion, confirming the formation of thermally stable crosslinked networks and the absence of significant degradation during vulcanization. Increasing carbon black loading progressively increased the final curing torque owing to enhanced compound viscosity and stronger filler networking. At the same loading of 1 phr, graphene generated the highest curing torque, followed by CNTs and carbon black, demonstrating that nanofiller geometry plays a decisive role in filler network formation during curing.

The reinforcing efficiency of the hybrid filler system depended on both filler content and morphology. Increasing carbon black loading enhanced stress at fixed elongation and tensile strength but reduced elongation at break above 10 phr because of increased stiffness and stress concentration. In contrast, graphene provided the most effective reinforcement among the investigated nanofillers, simultaneously yielding the highest tensile strength and elongation at break, thereby demonstrating the ability of two-dimensional nanostructures to improve mechanical performance without compromising deformability.

Partial replacement of silica with carbon black substantially reduced the Mullins effect compared with the silica-filled reference compound, although no monotonic relationship between carbon black loading and stress softening was observed. Conversely, graphene- and CNT-filled compounds exhibited greater stress softening than the carbon-black-filled formulation, suggesting that stronger polymer–filler interactions promote more pronounced stress-induced structural changes during cyclic deformation.

Dynamic mechanical analysis confirmed that the architecture of the reinforcing network is controlled by both filler chemistry and morphology. Increasing carbon black loading progressively weakened the strain-sensitive three-dimensional silica network, resulting in lower storage modulus values. At identical loading, carbon black generated the highest storage modulus and the strongest filler–filler interactions, whereas graphene and CNTs formed less rigid but mechanically more strain-tolerant networks. No synergistic enhancement of the filler network was observed within the investigated hybrid systems, indicating that silica remained the dominant contributor to filler networking. Since the Payne effect primarily reflects the strength of filler–filler interactions rather than filler dispersion, complementary microscopic characterization is required to establish the relationship between filler morphology and dispersion quality.

The thermal response of the investigated compounds was governed by the combined effects of heat transport and hysteretic energy dissipation. Although thermal conductivity generally increased with the incorporation of carbon nanofillers, thermographic analysis during cyclic De Mattia flexing demonstrated that self-heating could not be explained solely by thermal conductivity. Instead, heat build-up depended strongly on the composition of the hybrid filler system, with no monotonic relationship between carbon black loading and temperature rise. The compound containing 5 phr carbon black exhibited the highest temperature increase during cyclic loading, whereas the graphene-filled compound showed substantially lower self-heating despite its superior thermal conductivity. These findings suggest that the efficiency of mechanical energy dissipation, determined by the viscoelastic response of the hybrid filler network, may be an important factor influencing thermo-mechanical behaviour under cyclic deformation.

Dynamic ageing further highlighted the role of filler morphology in controlling structural stability. Increasing carbon black loading reduced the extent of sulfur bond rearrangement and additional crosslink formation, indicating enhanced network stability during cyclic loading. Intermediate carbon black contents (5–10 phr) provided the greatest resistance to hardness changes during ageing, whereas excessive carbon black loading promoted localized stress concentration and reduced hardness after prolonged fatigue. Among the investigated nanofillers, CNTs appeared to provide the greatest stabilization of the crosslink structure, whereas graphene may have promoted continued possible network reorganization and additional crosslink formation. The increase in hardness observed after prolonged ageing for graphene- and CNT-filled compounds was not directly correlated with changes in crosslink density, suggesting that filler–rubber interactions and local structural changes may also contribute to the long-term evolution of the elastomer network.

GC-IMS analysis provided direct chemical evidence of the degradation processes occurring during dynamic ageing and complemented the mechanical and thermal characterization. Cyclic loading promoted the formation of volatile degradation products, including benzothiazole and m-xylene, confirming that repeated deformation induces chemical changes within the elastomer network. The abundance of these volatile compounds strongly depended on the hybrid filler composition. The highest emission of degradation products was observed for the silica-rich formulations and for compounds containing low carbon black contents, whereas increasing the carbon black loading markedly reduced the intensity of the GC-IMS signals. The incorporation of graphene and carbon nanotubes resulted in the lowest abundance of volatile degradation products, indicating that carbon nanofillers effectively suppress thermo-mechanical degradation of the elastomer network. The agreement between the GC-IMS results and the mechanical, thermographic, and crosslink-density analyses demonstrates that volatile compound analysis is a valuable complementary tool for assessing degradation mechanisms in dynamically loaded rubber materials.

Overall, the present study demonstrates that the performance of hybrid silica/carbon-filled S-SBR compounds is governed by two complementary design parameters: the concentration of the carbon filler and its morphology. Carbon black primarily strengthens the filler network and improves static mechanical properties, graphene provides the most effective combination of reinforcement, thermal conductivity, and low heat build-up during cyclic loading, whereas carbon nanotubes appeared to exhibit the greatest ability to stabilize the elastomer network during dynamic ageing. More importantly, the integration of rheological, mechanical, dynamic mechanical, thermographic, crosslink-density, and GC-IMS analyses establishes a comprehensive structure–property–degradation relationship for hybrid silica/carbon-filled elastomers. This multi-technique approach provides new insight into the mechanisms governing reinforcement, energy dissipation, thermo-mechanical ageing, and chemical degradation, offering practical guidelines for the design of multifunctional rubber compounds for demanding dynamic applications, particularly advanced tire tread formulations requiring the simultaneous optimization of mechanical performance, thermal management, fatigue durability, and long-term structural stability.

The present results are consistent with previous studies demonstrating that carbon black primarily provides conventional mechanical reinforcement [15], graphene offers an outstanding combination of reinforcement and thermal transport [18,19], whereas carbon nanotubes are particularly effective in improving fatigue resistance and structural stability under cyclic loading [21,22]. However, unlike most previous studies, which focused mainly on mechanical or fatigue performance alone, the present work combines rheological, mechanical, thermo-mechanical, crosslink-density, and GC-IMS analyses to establish a comprehensive structure–property–degradation relationship for hybrid silica/carbon-filled S-SBR compounds.

## 5. Conclusions

This study demonstrates that the performance of hybrid silica/carbon-filled S-SBR compounds is governed by both the concentration and the morphology of the carbon filler, with filler geometry exerting a greater influence on network development and long-term behaviour than filler content alone. While carbon black primarily enhanced filler networking and static mechanical reinforcement, graphene provided the most favourable combination of mechanical performance, thermal conductivity, and reduced heat build-up during cyclic deformation. Carbon nanotubes, in turn, appeared to exhibit the highest ability to stabilize the elastomer network during thermo-mechanical ageing, leading to improved resistance to structural degradation.

The results further show that thermo-mechanical performance cannot be explained by thermal conductivity alone but is likely influenced by the efficiency of hysteretic energy dissipation within the hybrid filler network. Likewise, the evolution of the crosslink structure during cyclic ageing depends not only on crosslink density but also on filler–rubber interactions and filler morphology.

The combination of rheological, mechanical, dynamic mechanical, thermographic, crosslink-density, and GC-IMS analyses established a comprehensive structure–property–degradation relationship for hybrid silica/carbon-filled elastomers. In particular, GC-IMS proved to be a valuable complementary technique for monitoring degradation processes, providing direct chemical evidence that agrees well with the observed changes in mechanical performance and network stability.

Overall, these findings demonstrate that tailoring both the type and amount of carbon filler enables the design of multifunctional hybrid elastomer composites with improved reinforcement, thermal management, fatigue resistance, and long-term durability. The insights obtained provide useful guidelines for the development of advanced rubber compounds for demanding dynamic applications, particularly tire tread formulations requiring the simultaneous optimization of mechanical performance, thermo-mechanical stability, and resistance to degradation.

## Figures and Tables

**Table 1:** Compositions of the tested blends. Values are given in phr.

Component	CB0/Sil20	CB1/Sil19	CB5/Sil15	CB10/Sil10	CB20/Sil0	CNT1/Sil19	G1/Sil19
S-SBR	100	100	100	100	100	100	100
Sulfur	0.6	0.6	0.6	0.6	0.6	0.6	0.6
TBBS	4	4	4	4	4	4	4
ZnO	4	4	4	4	4	4	4
Stearic acid	4	4	4	4	4	4	4
Carbon filler	0	1	5	10	20	1	1
Silica	20	19	15	10	0	19	19

The names of the blends are as follows: CB—carbon black, Sil—silica, CNT—carbon nanotubes, G—graphene.

**Table 2:** Tensile strength results of carbon black-filled SBR compounds.

Sample Identification	SE100 [MPa]	SE200 [MPa]	SE300 [MPa]	TS [MPa]	Eb [%]
CB0/Sil20	0.51 ± 0.02	0.82 ± 0.05	1.22 ± 0.11	2.11 ± 0.19	476.3 ± 25.8
CB1/Sil19	0.73 ± 0.03	1.13 ± 0.10	1.65 ± 0.12	3.98 ± 0.21	615.4 ± 38.7
CB5/Sil15	0.79 ± 0.05	1.29 ± 0.09	1.95 ± 0.15	4.98 ± 0.43	564.2 ± 31.5
CB10/Sil10	0.91 ± 0.04	1.55 ± 0.07	2.41 ± 0.10	5.23 ± 0.31	525.0 ± 29.9
CB20/Sil0	1.04 ± 0.04	1.94 ± 0.08	3.14 ± 0.12	5.91 ± 0.36	460.9 ± 32.1

**Table 3:** Hysteresis results of carbon black-filled SBR compounds.

Sample Identification	W_1_	W_5_	Em [%]
CB0/Sil20	140.7 ± 8.7	92.7 ± 6.1	51.8 ± 11.3
CB1/Sil19	186.6 ± 9.1	151.6 ± 8.7	23.1 ± 9.3
CB5/Sil15	195.7 ± 9.5	159.7 ± 8.1	22.6 ± 8.7
CB10/Sil10	178.1 ± 9.1	139.4 ± 7.9	27.8 ± 10.0
CB20/Sil0	215.6 ± 9.9	175.0 ± 8.5	23.2 ± 8.3

**Table 4:** Temperature increase in the investigated SBR compounds during cyclic De Mattia fatigue.

Identification of the Specimen	Number of Cycles	Temperature Rise [°C]
CB0/Sil20	100	0.7 ± 0.1
	500	2.1 ± 0.3
	2000	1.7 ± 0.1
	10,000	2.4 ± 0.2
CB1/Sil19	100	0.2 ± 0.0
	500	0.3 ± 0.0
	2000	1.1 ± 0.1
	10,000	0.7 ± 0.1
CB5/Sil15	100	0.9 ± 0.1
	500	2.8 ± 0.3
	2000	2.6 ± 0.3
	10,000	4.5 ± 0.4
CB10/Sil10	100	0.9 ± 0.1
	500	2.5 ± 0.3
	2000	1.9 ± 0.2
	10,000	3.4 ± 0.3
CB20/Sil0	100	1.2 ± 0.1
	500	1.6 ± 0.2
	2000	3.6 ± 0.4
	10,000	3.9 ± 0.4

**Table 5:** Thermal conductivity of carbon black-filled SBR compounds with ± 0.005 measurement uncertainty.

Sample Identification	10 °C	20 °C	40 °C
CB0/Sil20	0.111 ± 0.002	0.112 ± 0.002	0.114 ± 0.002
CB1/Sil19	0.100 ± 0.001	0.103 ± 0.002	0.107 ± 0.002
CB5/Sil15	0.115 ± 0.002	0.116 ± 0.002	0.117 ± 0.002
CB10/Sil10	0.113 ± 0.002	0.114 ± 0.002	0.116 ± 0.002
CB20/Sil0	0.113 ± 0.002	0.113 ± 0.002	0.115 ± 0.002

**Table 6:** Tensile strength results of SBR compounds filled with different carbon fillers.

Sample Identification	SE100 [MPa]	SE200 [MPa]	SE300 [MPa]	TS [MPa]	Eb [%]
CB1/Sil19	0.73 ± 0.03	1.13 ± 0.08	1.65 ± 0.09	3.98 ± 0.16	615.4 ± 38.7
CNT1/Sil19	0.79 ± 0.04	1.19 ± 0.08	1.72 ± 0.10	3.96 ± 0.18	603.1 ± 35.1
G1/Sil19	0.87 ± 0.05	1.39 ± 0.09	2.04 ± 0.13	5.34 ± 0.25	667.6 ± 38.5

**Table 7:** Hysteresis results of SBR compounds filled with different carbon fillers.

Sample Identification	W_1_	W_5_	Em [%]
CB1/Sil19	186.6 ± 8.7	151.6 ± 7.1	23.1 ± 8.2
CNT1/Sil19	172.9 ± 7.2	134.7 ± 6.8	28.4 ± 8.8
G1/Sil19	198.5 ± 9.3	152.0 ± 7.5	30.7 ± 9.0

**Table 8:** Temperature increase in the investigated SBR compounds during cyclic De Mattia fatigue.

Identification of the Specimen	Number of Cycles	Temperature Rise [°C]
CB1/Sil19	100	0.2 ± 0.0
	500	0.3 ± 0.0
	2000	1.1 ± 0.1
	10,000	0.7 ± 0.1
G1/Sil19	100	0.2 ± 0.0
	500	1.4 ± 0.2
	2000	1.4 ± 0.1
	10,000	1.5 ± 0.2
CNT1/Sil19	100	0.3 ± 0.0
	500	3.4 ± 0.3
	2000	2.5 ± 0.2
	10,000	3.4 ± 0.3

**Table 9:** Thermal conductivity of SBR compounds filled with different carbon-based fillers at 1 phr loading with ±0.005 measurement uncertainty.

Sample Identification	10 °C	20 °C	40 °C
CB1/Sil19	0.100 ± 0.001	0.103 ± 0.001	0.107 ± 0.002
CNT1/Sil19	0.107 ± 0.002	0.107 ± 0.002	0.109 ± 0.002
G1/Sil19	0.124 ± 0.002	0.125 ± 0.002	0.126 ± 0.002

**Table 10:** Semi-quantitative GC–IMS parameters of the characteristic degradation signal detected in aged rubber compounds.

Compound	Max Height Range in Area	Area at Max Height Range	Ion Current Area
CB0/Sil20	1.788 ± 0.184	0.238 ± 0.030	4763.76 ± 1020.67
CB1/Sil19	1.892 ± 0.206	0.257 ± 0.021	5778.75 ± 641.72
CB5/Sil15	1.896 ± 0.006	0.255 ± 0.001	5674.90 ± 75.84
CB10/Sil10	1.824 ± 0.070	0.250 ± 0.012	5401.48 ± 387.16
CB20/Sil0	0.991 ± 0.222	0.129 ± 0.027	2491.80 ± 636.15
CNT1/Sil19	0.746 ± 0.275	0.099 ± 0.035	1348.44 ± 433.34
G1/Sil19	0.659 ± 0.034	0.086 ± 0.008	1405.10 ± 281.65

## Data Availability

The original contributions presented in this study are included in the article. Further inquiries can be directed to the corresponding author.
